# EMT is associated with an epigenetic signature of ECM remodeling genes

**DOI:** 10.1038/s41419-019-1397-4

**Published:** 2019-02-27

**Authors:** Paul Peixoto, Amandine Etcheverry, Marc Aubry, Anaïs Missey, Camille Lachat, Jérôme Perrard, Elodie Hendrick, Régis Delage-Mourroux, Jean Mosser, Christophe Borg, Jean-Paul Feugeas, Michaël Herfs, Michaël Boyer-Guittaut, Eric Hervouet

**Affiliations:** 1 Univ. Bourgogne Franche-Comté, INSERM, EFS BFC, UMR1098, Interactions Hôte-Greffon-Tumeur/Ingénierie Cellulaire et Génique, F-25000 Besançon, France; 2 EPIGENEXP Platform, University of Bourgogne Franche-Comté, F-25000 Besançon, France; 3 CHU Rennes, Service de Génétique Moléculaire et Génomique, F-35033 Rennes, France; 4 Plate-forme Génomique Environnementale et Humaine Biosit, Université Rennes1, F-35043 Rennes, France; 5 Cancéropole Grand-Ouest, Réseau Epigénétique (RepiCGO), Rennes, France; 6 CNRS, UMR 6290, Institut de Génétique et Développement de Rennes (IGDR), F-35043 Rennes, France; 7 Faculté de Médecine, Université Rennes1, UEB, UMS 3480 Biosit, F-35043 Rennes, France; 8 Laboratory of Experimental Pathology, GIGA-Cancer, University of Liege, 4000 Liege, Belgium; 9 Department of Medical Oncology, University Hospital of Besançon ou Hôpital Universitaire de Besançon, F-25000 Besançon, France; 10 INSERM CIC-1431, University Hospital of Besançon ou Hôpital Universitaire de Besançon, Clinical Investigation Center in Biotherapy, F-25000 Besançon, France; 11 DimaCell Platform, Univ. Bourgogne Franche-Comté, F-25000 Besançon, France

## Abstract

Type III epithelial–mesenchymal transition (EMT) has been previously associated with increased cell migration, invasion, metastasis, and therefore cancer aggressiveness. This reversible process is associated with an important gene expression reprogramming mainly due to epigenetic plasticity. Nevertheless, most of the studies describing the central role of epigenetic modifications during EMT were performed in a single-cell model and using only one mode of EMT induction. In our study, we studied the overall modulations of gene expression and epigenetic modifications in four different EMT-induced cell models issued from different tissues and using different inducers of EMT. Pangenomic analysis (transcriptome and ChIP–sequencing) validated our hypothesis that gene expression reprogramming during EMT is largely regulated by epigenetic modifications of a wide range of genes. Indeed, our results confirmed that each EMT model is unique and can be associated with a specific transcriptome profile and epigenetic program. However, we could select some genes or pathways that are similarly regulated in the different models and that could therefore be used as a common signature of all EMT models and become new biomarkers of the EMT phenotype. As an example, we can cite the regulation of gene-coding proteins involved in the degradation of the extracellular matrix (ECM), which are highly induced in all EMT models. Based on our investigations and results, we identified ADAM19 as a new biomarker of in vitro and in vivo EMT and we validated this biological new marker in a cohort of non-small lung carcinomas.

## Introduction

Type III epithelial–mesenchymal transition (EMT) is a reversible process that contributes to invasion and metastasis. EMT is characterized by a downregulation of epithelial markers and an increase in mesenchymal markers and in EMT-linked transcription factors^1^, but the molecular mechanisms governing EMT remain poorly understood. For the past decade, the role of epigenetics in EMT regulation has clearly emerged. For example, the histone methyl transferase (HMT) EZH2 was required to downregulate *miR211* and EMT induction in glioblastoma multiforme^2^, but, on the opposite, the histone demethylase KDM6B induced SNAIL2 and EMT^3^. The interaction of the transcription factor TWIST with another HMT, KMT5/SET8, has also been associated with the repression of *CDH1*^4^. Although these publications strongly support a role for epigenetic modifications during EMT, they all have been described in the same subtype of cancer or a single EMT cell model. Moreover, the high number of EMT markers described from one model to another suggest that EMT is a complex mechanism linked to different signaling pathways^5^ that led us to the hypothesis of the existence of numerous tissue- or cell-specific EMT.

To further characterize the epigenetic mechanisms regulating EMT, we decided to study overall gene expression and their associated epigenetic modifications in several EMT models. To do so, we used the A549 (non-small cell lung cancer (NSCLC)), the ACHN (renal cell carcinoma derived from pleural effusion), and the immortalized breast MCF10A cell lines treated, or not, with transforming growth factor beta (TGFβ)/tumor necrosis factor alpha (TNFα) to induce EMT^6^. To confirm that the observed effects were not linked to the use of this combination of cytokines, we also used the breast cancer (BC) MDA-MB-468 cells treated, or not, with epidermal growth factor (EGF)^7^. As expected, using microarray and chromatin immunoprecipitation (ChIP)–sequencing (ChIP-seq) analyses, we showed that EMT-associated genes were largely regulated by epigenetic modifications. Our results also demonstrated, for the first time, that cell origin and EMT inducer were associated with a specific cellular response. However, the regulation of genes involved in the degradation of the extracellular matrix (ECM), in particular *ADAM19* (ADAM Metallopeptidase Domain 19) coding a metallopeptidase, was strongly regulated by epigenetics during EMT, independently of the EMT model, and we showed that epigenetic modifications were crucial for EMT in these cancer models.

## Results

### EMT-induced cell models

**Fig. 1:**
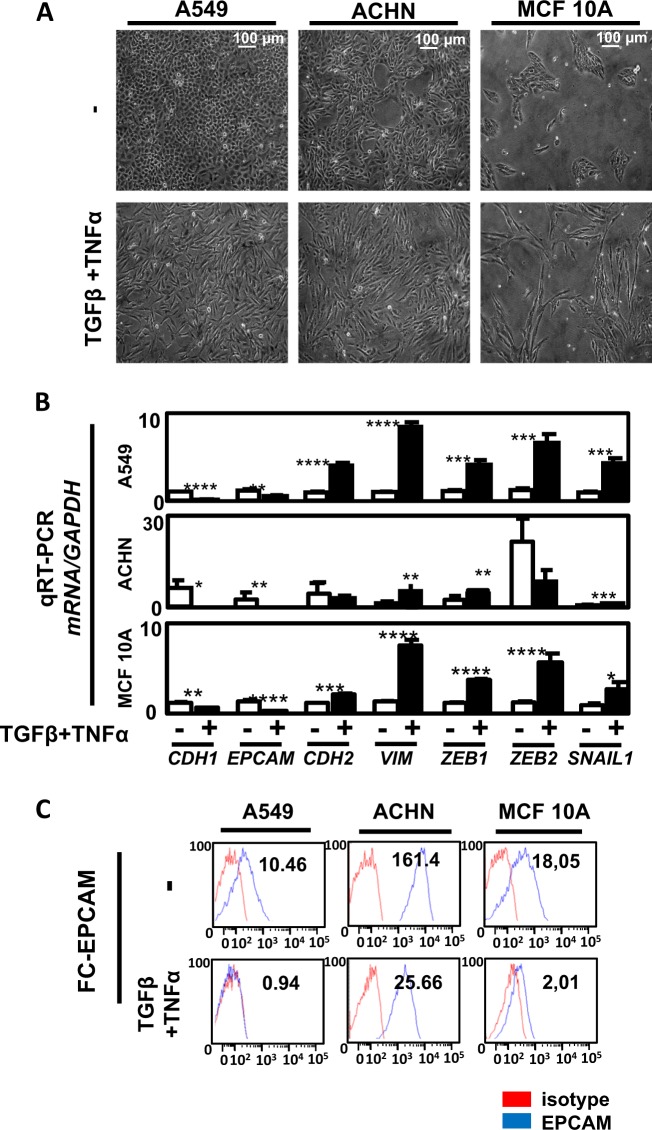
Transforming growth factor beta (TGFβ)/tumor necrosis factor alpha (TNFα) treatment induced epithelial–mesenchymal transition (EMT) in the A549, ACHN, and MCF10A models. **a** A549, ACHN, and MCF10A cells were seeded in 6-multiwell dishes and treated for 5 days with TGFβ and TNFα. The pictures presented are representative of at least three independent experiments. **b** Expression of epithelial gene markers (*CDH1* and *EPCAM*), mesenchymal gene markers (*CDH2*, *VIMENTIN*) and EMT-linked transcription factors (*ZEB1*, *ZEB2*, and *SNAI1*) were measured by quantitative reverse transcriptase–polymerase chain reaction (independent triplicates) in cells treated with or without TGFβ/TNFα for 5 days (white bars: untreated; black bars: TGFβ/TNFα). **c** A decrease of the epithelial marker EPCAM was confirmed using flow cytometry in the 3 cell lines following treatment with TGFβ/TNFα for 5 days

Our initial goal was to study the effects of EMT inducers on cells issued from different organs (lung, kidney, and breast). We then treated A549, ACHN, and MCF10A cells with TGFβ/TNFα. TGFβ is a well-admitted EMT inducer and TNFα has been described to potentiate EMT induction by stabilizing SNAIL in a nuclear factor-κB-dependent manner^8,9^. Following induction, a strong mesenchymal-like phenotype with the loss of cell–cell adhesion and an increase in cell elongation occurred (Fig. 1a). We next observed using quantitative reverse transcriptase–polymerase chain reaction (qRT-PCR) that EMT markers were induced in the three cell lines but the fold changes were dependent of the cell line (Fig. 1b). We indeed quantified a strong decrease in epithelial markers *CDH1* and *EPCAM* and an increase in mesenchymal markers *SNAI1*, *VIM*, *ZEB1*, and *ZEB2* in the three models but the increase in *VIM*, *ZEB1*, and *ZEB2* expression was higher in A549 and MCF10A cells compared to the ones observed in the ACHN cells, suggesting that EMT induction is, at least partially, different in regard to cell origin. Regarding *MMP9* mRNA expression, an increase was observed in the three cell lines during EMT (Supp Fig. 1A/C). These data were confirmed at the protein level since we detected a strong decrease in EPCAM expression in these cell lines using flow cytometry (Fig. 1c) and a significant decrease in E-CADHERIN levels together with an increase of VIMENTIN in A549 and ACHN cells using western blotting (WB) and immunofluorescence (IF) (Supp Fig. 1B; Supp Fig. 1C).

### Transcriptome analysis of EMT-induced models

**Fig. 2:**
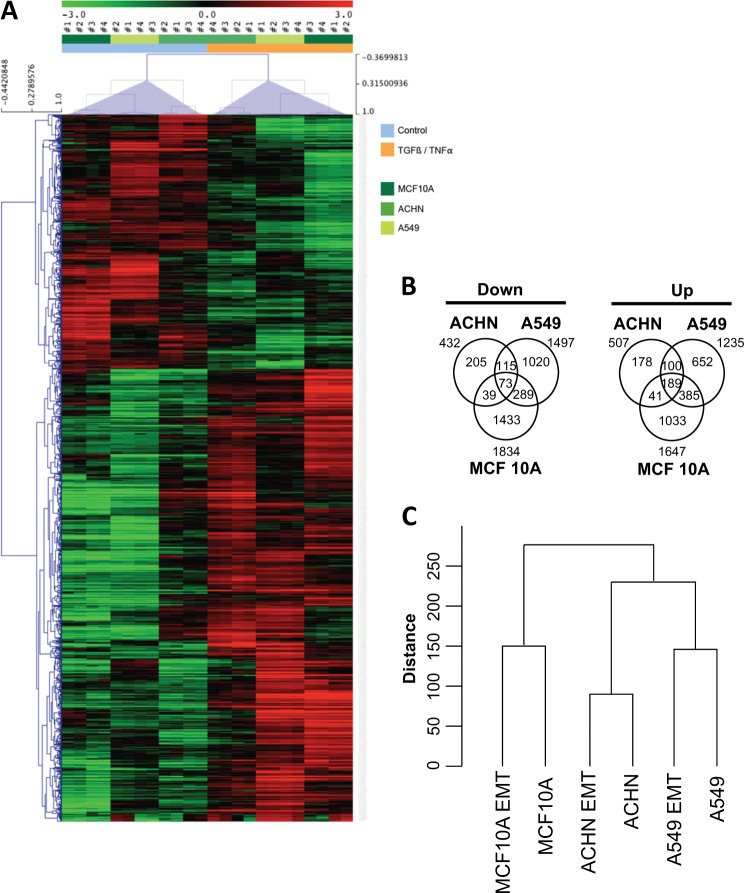
Transcriptome analysis of genes regulated during epithelial–mesenchymal transition induction **a** Gene expression was quantified by microarray in A549, ACHN, and MCF10A cells treated with or without transforming growth factor beta/tumor necrosis factor alpha for 5 days (*n* = 4). **b** Venn diagrams showing the distribution of upregulated and downregulated genes in the three cell lines. **c** Cluster dendrogram of the different transcriptomes

Next, we decided to compare the EMT-linked gene expression in A549, ACHN, and MCF10A cell lines using microarray. High (red) or low decreased (green) gene expression was classified in regard to the cell line model and TGFβ/TNFα treatment (Fig. 2a) (*n* = 4). In the A549 cells, 3636 probes were differentially expressed (DE) in treated versus control conditions (1763 upregulated and 1873 downregulated). In the ACHN cells, 1141 probes were DE (624 upregulated and 517 downregulated). In the MCF10A cells, 4683 probes were DE (2092 upregulated and 2591 downregulated) (Supp Table 1). Gene Set Enrichment Analysis (GSEA) performed on the 258 TGFβ-/TNFα-regulated genes identified activated canonical pathways related to ECM organization and remodeling, Beta1 integrin cell surface interactions, and focal adhesion (Supp Table 1). A list of the 30 genes presenting the highest fold change expression within the three cell lines is reported in Table 1. Among these genes, the expression of *MMP1*, *MMP9*, *MMP10*, *ADAM19*, or *ADAMTS6*, a family of proteins involved in ECM remodeling^10^, presented a 126-, 114-, 43-, 27-, and 34-fold increase, respectively. On the opposite, the expression of *CDH1* (41-fold decrease) was 1 of the only 2 genes downregulated among our selected list of 30 (Table 1). The induction of matrix metalloproteinase (MMP) protein levels (intracellular MMP9 by WB) and its activity (zymography) were confirmed in the A549 model (significant increase in excreted MMP2 (*p* = 0.002) and MMP9 (*p* = 0.016); Supp Fig. 2). Altogether, these data validated our EMT models as well as our protocol design to search for new regulated genes during EMT.

**Table 1:** List of the 30 genes with the most important modification of expression after TGFβ and TNFα treatment

Gene	*p* Value	Up/down	Fold	Protein type/RNA	Pathways
CCL5	4.2E−9	Up	131.8	Chemokine	Immunoregulation
IL7R	9.7E−10	Up	149.4	Interleulin receptor	Immunoregulation
MMP1	3.5E-1	Up	126	Matrix metallopeptidase	Invasion
MMP9	2.2E−4	Up	114.1	Matrix metallopeptidase	Invasion
C4orf26	1.0E−4	Up	100.1	Extracellular protein	Mineralization
UBD	1.4E−5	Up	105.8	Ubuiquitin	Protein ubiquitination/TNF pathway
LCE3D	0.0039	Up	99.2	Late cornified envelope	Keratinization/development
INHBA	0.0040	Up	82.6	FSH secretion inhibitor	Growth/differentiation factor
CSF2	0.0070	Up	66.5	Cytokine	Immunoregulation
SLAMF8	3.9E−6	Up	60.4	Cell surface receptor	Immunoregulation
ROBO4	2.0E−7	Up	62.4	Cell surface receptor	Angiogenesis/migration
MROH3P	1.5E−6	Up	40.0	pseudogene	
CDH1	0.0011	Down	40.8	Cadherin	Cell adhesion
MMP10	1.5E−5	Up	42.6	Metalloprotease	Invasion/metastasis
FAP	0.00389	Up	45.1	Cell surface protein	Invasion/immunoregulation/tumor growth
lnc-C15orf48–1	1.9E−6	Up	45.2	Non-coding RNA	
C15orf48	0.0012	Up	47.0		Metabolism
lnc-LRRC1–5	1.3E−5	Up	47.7	Non-coding RNA	
LAMC2	2.1E−5	Up	47.8	Extracellular protein	Adhesion/differentiation/migration/metastasis
FGFBP1	0.0014	Down	48.3	Extracellular protein	Cell proliferation/diffentiation/migration
NLRP3	0.0039	Up	30.9	Cell signaling	Inflammation/apoptosis
BMP2	0.0013	Up	33.6	Cytokine	Differentiation
KANK4	0.0060	Up	34.0	Cell signaling	Cytoskeleton remodeling
ADAMTS6	9.3E−5	Up	34.2	Metallopeptidase	Migration/Invasion
DHRS2	2.0E−6	Up	38.9	Dehydrogenase/reductase	
DCN	7.0E−5	Up	39.7	Proteoglycan	Migration /TGFb signaling
LCE3E	0.0016	Up	29.8	Late cornified envelope	Keratinization/development
CXCL8	7.3E−5	Up	29.6	Chemokine	Inflammation
LINC01583	6.2E−6	Up	28.1	Non-coding RNA	
ADAM19	9.3E−7	Up	27.4	Metallopeptidase	Adhesion

List of 30 genes issued from microarrays analysis with the most important fold increase or decrease expression. All transcriptome profiles were simultaneously analyzed (16 NT (A549 NT *n* = 4, ACHN NT *n* = 4, MCF10A cells NT *n* = 4) versus16 TGFβ/TNFα treated 16 NT (A549 treated *n* = 4, ACHN treated *n* = 4, MCF10A cells treated *n* = 4). Name of the gene, *p* value, fold change, and molecular-associated mechanisms (except for genes with unknown functions) are indicated.

*TGFβ* transforming growth factor beta, *TNFα* tumor necrosis factor alpha

We also confirmed (Fig. 1) that morphological modifications were related to EMT in our models, and then the differential expression of the 12 main EMT markers was quantified in each cell line (Table 2). As expected and described above, *CDH1* expression was highly significantly decreased in the three cell lines following TGFβ/TNFα treatment and similar results were obtained for *EPCAM*, but the expression of other EMT markers was not consistent throughout the three cell lines. For example, *ZEB1* expression was increased in both A549 and MCF10A cells but stable in ACHN cells (Table 2). These data strongly suggested that different and independent molecular signaling pathways might regulate different model-linked EMT. We then established Venn diagrams describing the distribution of upregulatedand downregulated genes and we observed that only 73 genes were concomitantly downregulated while 189 were concomitantly upregulated in the 3 cell lines during EMT (Fig. 2b), with most of the dysregulated genes (up or down) modified in only one or two models. Unsupervised hierarchical clustering analysis of the probes displaying the greatest variation among the samples (standard deviation) showed that transcriptomes were first clustered by cell line and then by TGFβ/TNFα treatment, confirming that differences in transcriptome profiles were primarily due to cell origin and secondarily to EMT induction (Fig. 2c).

**Table 2:** Expression of genes related to EMT after TGFβ and TNFα treatment

Gene	Protein	Cells	*p* Value	Up/down	Fold	Marker
CDH1	E-CADHERIN	A549	1.6E−6	Down	250	Epithelial
		ACHN	0.007	Down	105	
		MCF10A	8.1E−4	Down	2.6	
EPCAM	EPCAM	A549	0.001	Down	14	Epithelial
		ACHN	0.001	Down	2.8	
		MCF10A	1.7E−4	Down	32	
CDH2	N-CADHERIN	A549	3.9E−4	Up	5.2	Mesenchymal
		ACHN	0.001	Up	2.0	
		MCF10A	1.8E−4	Up	6.3	
VIM	VIMENTIN	A549	0.003	Up	2.0	Mesenchymal
		ACHN	—			
		MCF10A	—			
ZEB1	ZEB1	A549	1.6E−4	Up	2.6	EMT-ATF
		ACHN	—			
		MCF10A	0.001	Up	3.8	
ZEB2	ZEB1	A549	6.3E−4	Up	2.8	EMT-ATF
		ACHN	—			
		MCF10A	0,001	Up	2.8	
SNAI1	SNAIL1	A549	3.1E−5	Up	2.8	EMT-ATF
		ACHN	—			
		MCF10A	2.0E-4	Up	7.0	
		A549	0.0010	Down	2.4	Epithelial
OCLN	OCCUDIN	ACHN	—			
		MCF10A	—			
ETS1	ETS1	A549	2.1E−5	Up	3.2	Mesenchymal
		ACHN	0.0024	Up	2.5	
		MCF10A	2.7E−5	Up	11.85	
FN1	FIBRONECTIN	A549	2.2E−4	Up	11.7	Mesenchymal
		ACHN	—			
		MCF10A	0.0091	Up	68.4	
SDC1	SYNDECAN-1	A549	3.0E−5	Up	2.8	Mesenchymal
		ACHN	—			
		MCF10A	—			
SNAI2	SLUG	A549	3.5E−6	Up	17.6	EMT-ATF
		ACHN	—			
		MCF10A	—			

List of genes issued from microarrays analysis (*n* = 4) in A549, ACHN, or MCF10A cells. *p* Value and fold change are indicated for each cell line. Epithelial markers (*CDH1*, *EPCAM*, *OCLN*, *SDC1*), mesenchymal markers (*CDH2*, *VIMENTIN*, *ETS1*, *FN1*), and EMT-ATFs (*ZEB1*, *ZEB2*, *SNAI1*, *SNAI2*) were analyzed

*EMT* epithelial–mesenchymal transition, *TGFβ* transforming growth factor beta, *TNFα* tumor necrosis factor alpha

To determine whether these different EMT phenotypes were dependent of the time of TGFβ/TNFα treatment, we performed a kinetic analysis from 24 to 120 h treatment with TGFβ/TNFα or each cytokine alone in A549 and ACHN cells (Supp Fig. 3). As expected, a progressing mesenchymal-like phenotype was observed along the course of treatment (Supp Figs. 3A and 3B) but the phenotype was the most pronounced with both cytokines. A progressive increase in *VIMENTIN*, *ZEB1*, and *SNAI1* and decrease in *CDH1* and *EPCAM* gene expression levels were observed during the treatment and were further increased with TGFβ/TNFα compared to each cytokine alone (Supp Fig. 2C and Supp Fig. 4A). A VIMENTIN IF confirmed these data at the protein level and it was more pronounced in A549 cells compared to ACHN (Supp Fig. 4B) and with the double treatment (data not shown).

### Global modulation of epigenetic marks during EMT

**Fig. 3:**
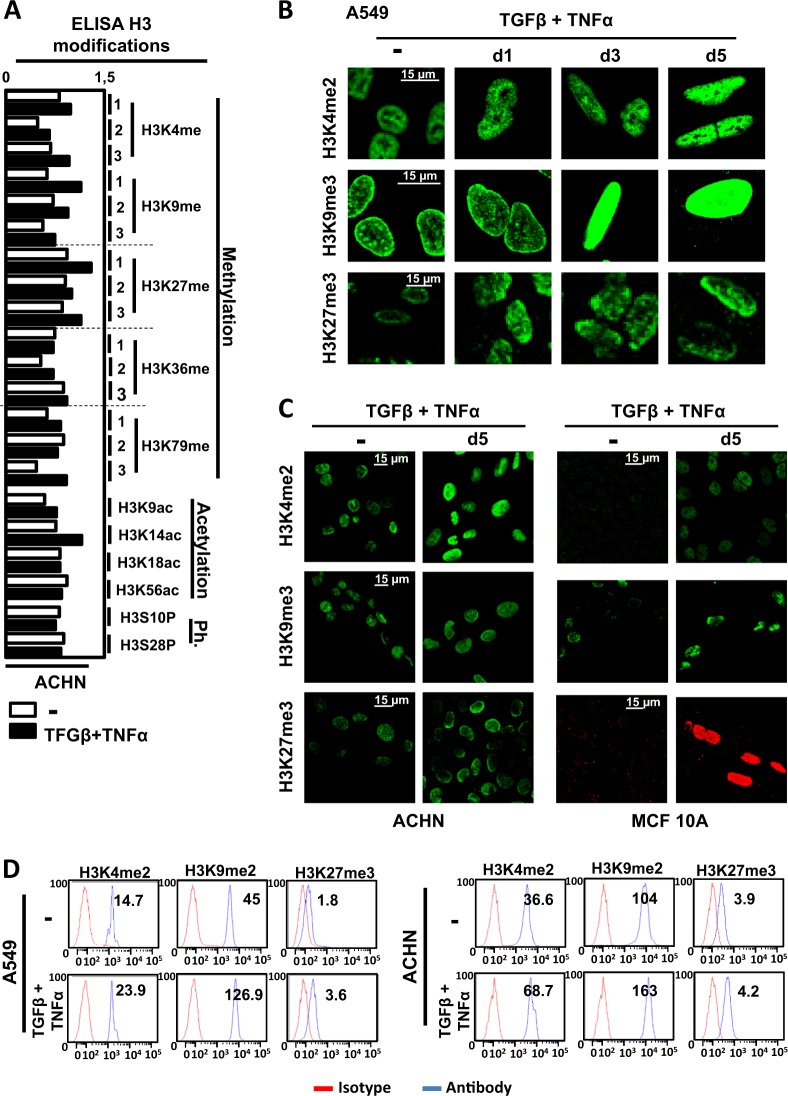
Transforming growth factor beta (TGFβ)/tumor necrosis factor alpha (TGFβ/TNFα) treatment regulates histone H3 modifications. **a** Histones were purified from ACHN cells treated with or without TGFβ/TNFα for 5 days. Twenty-one posttranslational modifications of histone H3 (acetylation: H3K9ac, H3K14ac, H3K18ac, H3K56ac; methylation: H3K4me1, H3K4me2, H3K4me2, H3K9me1, H3K9me2, H3K9me3, H3K27me1, H3K27me2, H3K27me3, H3K36me1, H3K36me2, H3K36me3, H3K79me1, H3K79me2, H3K79me3; and phosphorylation: H3S10P, H3S28P) were quantified using a multiplex enzyme-linked immunosorbent assay. Values were normalized to total H3 content in the same measurement. Each bar represents the mean of two independent measurements and each one was obtained from a mix of two independent histone extractions. **b**, **c** Increase staining of H3K4me2, H3K9me3, and H3K27me3 marks observed using immunofluorescence in A549, ACHN, and MCF10A cells treated with or without TGFβ/TNFα for 5 days. Each picture is representative of a typical result from at least three independent experiments. **d** Increased staining in H3K4me2, H3K9me3, and H3K27me3 marks quantified using flow cytometry in A549, ACHN, and MCF10A cells treated with or without TGFβ/TNFα for 5 days (representative results of at least 3 independent experiments)

We next wondered whether epigenetic modifications occurred in these cells and whether the epigenetic mechanisms were similar in these different models. To do so, we used an enzyme-linked immunosorbent assay protocol to quantify 21 posttranslational H3 modifications in ACHN cells treated, or not, with TGFβ/TNFα (Fig. 3a). This experiment suggested that global histone methylation of H3K4, H3K9, and H3K27 were highly modified during EMT, while only low changes were observed for acetylation or phosphorylation of H3, apart from H3K14ac. We then performed IF (Fig. 3b, c) and flow cytometric (Fig. 3d) analyses of global H3K4me2, H3K9me3, and H3K27me3 in the three cell lines following TGFβ/TNFα treatment and indeed confirmed an important increase in these different marks during EMT. These strong global histone modifications observed during EMT suggested that epigenetics might play a pivotal role in transcription reprogramming during EMT.

### Genes involved in ECM degradation were regulated by epigenetics in TGFβ-/TNFα-treated cells

**Fig. 4:**
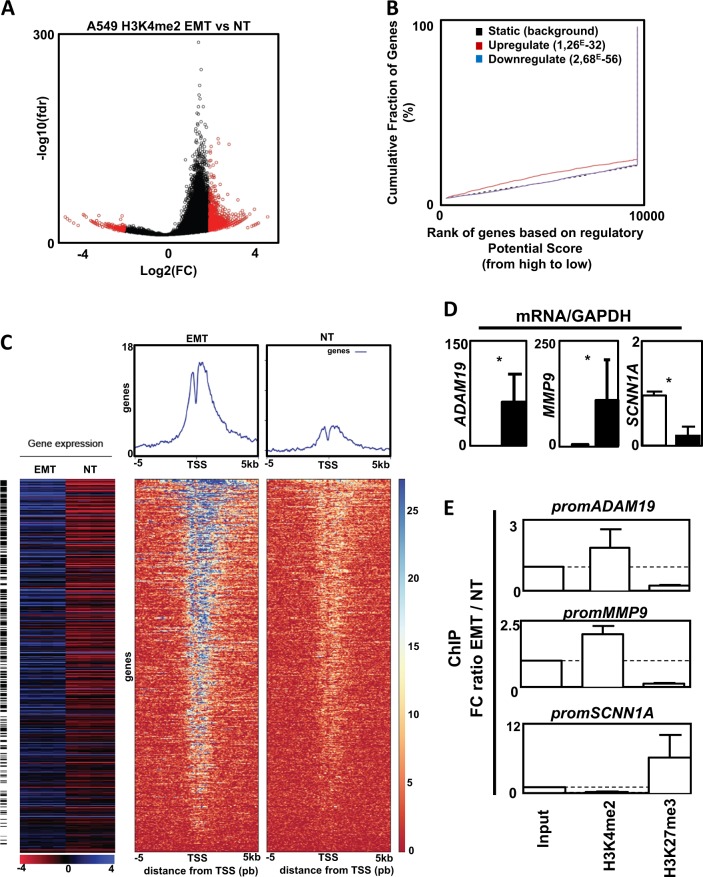
Chromatin immunoprecipitation (ChIP) and ChIP-seq analysis on the H3K4me2 mark following transforming growth factor beta (TGFβ)/tumor necrosis factor alpha (TGFβ/TNFα) treatment. **a** Volcano plot of the 42,076 H3K4me2 merged islands. In red, the regions significantly enriched in TGFβ-/TNFα-treated cells versus non-treated cells. FC: fold change treated versus non-treated. fdr: false discovery rate treated versus non-treated. **b** Activating and/or repressive function prediction of H3K4me2 in A549 cells. BETA-basic integrates H3K4me2 differentially enriched regions and transcriptome data on TGFβ/TNFα treated cells and non-treated conditions to identify upregulated (red) and downregulated (purple) genes. The dashed line indicates the non-differentially expressed genes as background. Genes are cumulated by the rank on the basis of the regulatory potential score from high to low. *p* Values represent the significance of difference in the upregulated or downregulated groups compared with the non-differentially expressed group by Kolmogorov–Smirnov test. **c** Integration of transcriptome and ChIP-seq data for the A549 cell line. Top: heat maps of read coverage from −5 kb to +5 kb around the transcription start site for TGFβ-/TNFα-treated (left) and non-treated (right) conditions. Each line represents an upregulated gene identified by BETA. Genes are ordered according to their increasing rank product. Upper panels: read coverage density plots. Bottom: Corresponding gene expression heat map for treated (*n* = 4) and non-treated conditions (*n* = 4). Black lines indicate differentially expressed genes. **d** Increase expression of ADAM19 and MMP9 and decrease expression of SCNN1A in A549 cells treated with TGFβ/TNFα (mean ± SD of at least 3 independent experiments). **e** Ratio of fold change (epithelial–mesenchymal transition: TGFβ/TNFα treated A549 versus NT: untreated A549 cells) following ChIP against H3K4me2 and H3K27me3 marks on *MMP9*, *ADAM19*, and *SCNN1A* promoters. Dotted line = 1 (mean ± SEM of at least 3 independent experiments) *p < 0.05

Since >90% of the most DE genes in our models were overexpressed during EMT, we decided to conduct ChIP-seq analyses in A549 cells treated, or not, with TGFβ/TNFα to target the H3K4me2 mark. Then we crossed our ChIP-seq data with the transcriptomes (Fig. 4**)**. We detected 1952 H3K4me2 regions that were significantly enriched in TGFβ-/TNFα-treated cells versus non-treated cells (1740 upregulated and 212 downregulated) (Fig. 4a and Supp Table 3), with most of these H3K4me2 regions located in introns (46%) (Supp Fig. 5). BETA analysis predicted an activating function of the H3K4me2 mark with a cumulative fraction of genes significantly above background for upregulated genes (Fig. 4b) and indeed identified 614 upregulated genes potentially activated by the H3K4me2 mark (Fig. 4a, Supp Table 4). Regarding these genes, the H3K4me2 mark was predominantly enriched in introns (81%) (Supp Fig. 5). Among the most significantly activated canonical pathways, identified by GSEA, we once again found genes linked to ECM organization, Beta1 integrin cell surface interactions, and focal adhesion (Supp Table 3). Among the overexpressed genes identified in the transcriptome analysis, 23% were found in the 614 genes potentially activated by the H3K4me2 mark.

To confirm that the expression of genes involved in ECM were indeed linked to epigenetic modifications during EMT, we selected *ADAM19* (22.6-, 4.5-, and 30.7-fold increase in A549, ACHN, and MCF10A, respectively) and *MMP9* (187- and 1000-fold increase in A549 and MCF10A, respectively, but no change in ACHN). The *SCNN1A* gene (12- and 4.5-fold decrease in A549 and MCF10A, respectively, but no change in ACHN cells) was used as a negative control. First, we confirmed a progressive modulation of *ADAM19*, *MMP9* and *SCNN1A* expression during EMT (Fig. 4d and Supp Fig. 6A). Next, we confirmed the epigenetic regulation during EMT using ChIP analysis (Fig. 4e). TGFβ/TNFα-induced EMT was clearly associated with an increase in the H3K4me2 mark on the promoters of *ADAM19* and *MMP9* and a decrease on the *SCNN1A* promoter. Moreover, ChIP targeting the repressive mark H3K27me3 showed opposite profiles on these promoters (Fig. 4e). We also established the progressive modification of these epigenetic marks during TGFβ/TNFα treatment as illustrated by kinetic ChIP experiments (Supp Fig. 6B). Since MCF10A cells are unable to form tumors in vivo, we also confirmed the induction of *MMP9* and *ADAM19* expression in MDA-MB-157 induced in EMT (Supp Figs. 7A and 7B). Moreover, no induction of these genes was observed in MCF-7, which failed to initiate EMT, whereas a strong expression of both *MMP9* and *ADAM19* was quantified in the mesenchymal MDA-MB-231 cells.

### The epigenetic regulation of genes involved in ECM degradation was independent of the EMT inducer

**Fig. 5:**
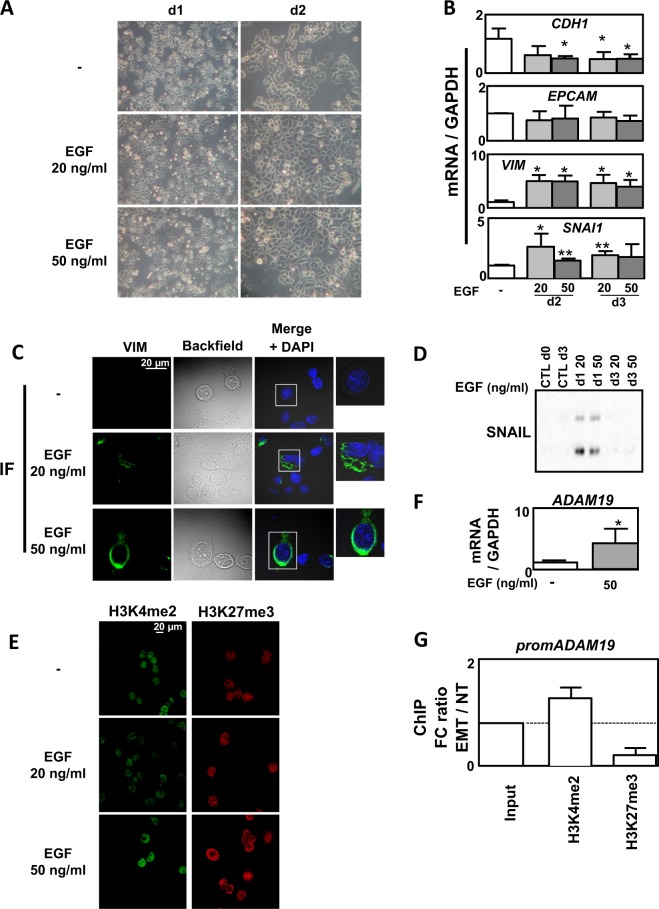
Epigenetic regulation in epidermal growth factor (EGF)-induced epithelial–mesenchymal transition (EMT) in MDA-MB-468 cells. **a** Representative images showing a change in morphology of MDA-MB-468 cells following 1–5 days of treatment with EGF (20 or 50 ng/ml). **b** Validation of EMT markers by quantitative reverse transcriptase–polymerase chain reaction (qRT-PCR) in the MDA-MB-468 cell line treated or not with EGF (20 or 50 ng/ml) for 3 days (mean ± SD of at least 3 independent experiments). **c** Increased change of morphology and intensity of VIMENTIN staining using immunofluorescence (IF) in MDA-MB-468 cells treated or not with EGF (20 or 50 ng/ml) for 3 days. **d** Increased expression of SNAIL1 in MDA-MB-468 cells treated or not with EGF (20 or 50 ng/ml) for 3 days. **e** Increased staining in H3K4me2 and H3K27me3 marks using IF in MDA-MB-468 cells treated or not with EGF (20 or 50 ng/ml) for 3 days (representative pictures of 3 independent experiments). **f** Increased expression of *ADAM19* gene quantified by qRT-PCR in MDA-MB-468 cells treated or not with EGF (50 ng/ml) for 3 days. **g** Ratio of fold change (EMT: EGF treated versus NT: untreated MDA-MB-468 cells) following chromatin immunoprecipitation against H3K4me2 and H3K27me3 marks on the *ADAM19* promoter. Dotted line = 1 (mean ± SEM of at least 3 independent experiments) *p < 0.05 and **p < 0.01

Even if we already used three different EMT models, we next wondered whether these results could be reproduced in a different and independent model to answer the fact that our observations might only be dependent of the TGFβ/TNFα treatment. We chose the epithelial MDA-MB-468 breast cells in which EMT can be induced using EGF^7^. After 2 days of EGF treatment, an important proportion of cells presented an increased tubular shape (Fig. 5a) together with an increase in the expression of *VIMENTIN* and *SNAI1* and a decrease in *CDH1* (Fig. 5b). EMT was also confirmed by increased VIMENTIN staining in IF (Fig. 5c) and increased SNAIL-1 levels in WB (Fig. 5d). We also confirmed a global increase in H3K4me2 and H3K27me3 in MDA-MB-468 treated with EGF (Fig. 5e). We next quantified the expression of our ECM remodeling genes in this new model and described that *ADAM19* expression was upregulated following EGF treatment (Fig. 5f). Moreover, this upregulation was correlated to an increase in the H3K4me2 mark on the *ADAM19* promoter together with a decrease in the H3K27me3 mark (Fig. 5g).

### In vivo confirmation of the regulation of genes involved in ECM degradation in EMT

**Fig. 6:**
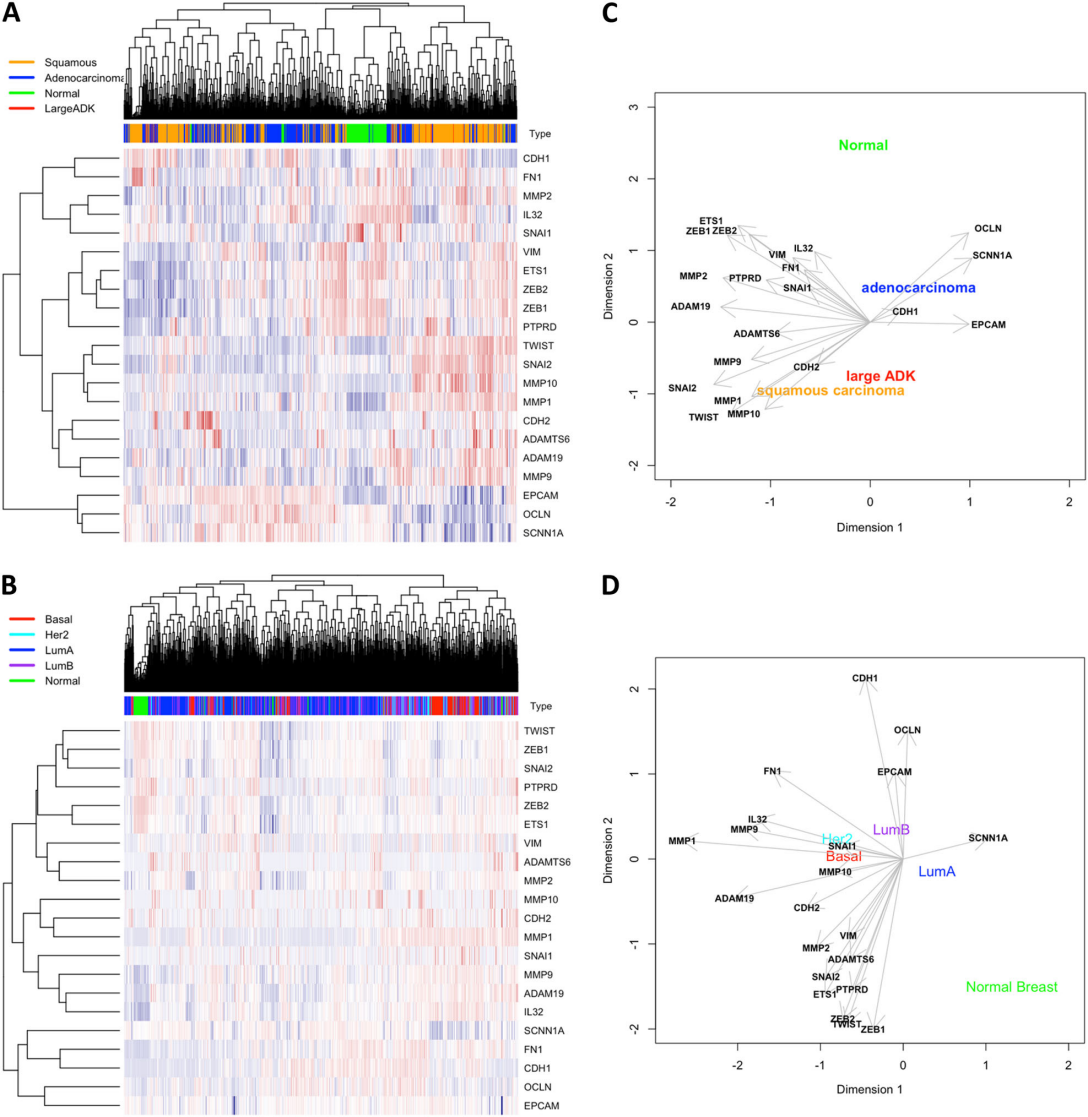
Large retrospective transcriptome analysis in non-small cell lung cancer (NSCLC) or breast cancer (BC) subtypes. Expression of *MMP9*, *ADAM19*, and *SCNN1A* mRNA was correlated to epithelial–mesenchymal transition (EMT) markers in a retrospective analysis using published microarrays. Expression of *MMP9*, *ADAM19*, *SCNN1A* was correlated to EMT markers in a retrospective analysis using microarrays downloaded from public datasets. Heat maps were drawn by hierarchical clustering of gene expression in different samples from lung (**a**) and breast (**c**) including primary tumors and normal tissue (“Normal”). High transcript levels are marked in red and low levels are marked in blue. Correlations between these markers were plotted in two dimensions after principal component analysis for NSCLC (**b**) and for BC subtypes (**d**)

To address the question whether the genes involved in the degradation of the ECM degradation was associated with EMT and aggressive subtypes in vivo, we performed a retrospective analysis using previously published transcriptomes issued from NSCLC microarrays (Fig. 6a). We also added the expression of EMT markers as well as the expression of the genes of the ECM degradation signature identified in A549 (upregulated: *ADAM19*, *ADAMTS6*, *MMP-1*, *MMP-2*, *MMP-10*, *MMP-9*, *IL32*, downregulated: *SCNN1A*) on this graph. (Fig. 6a, c). As expected, large ADK or squamous lung cancers, which are associated with higher risks of metastasis compared to adenocarcinoma, presented a high expression of the ECM degradation genes and a low expression of epithelial markers compared to normal tissues. These data confirmed that ECM degradation biomarkers induced in our EMT models were associated with EMT and aggressiveness in vivo. Since we also confirmed the ECM degradation signature in our BC models, we analyzed 2627 transcriptomes from BC (Fig. 6b, d). The different subtypes, Luminal A, Her2, Basal, or normal breast tissues, were plotted on a three-dimensional diagram based on the Prediction Analysis of Microarray 50 (PAM50) classification using *ESR1*, *HER2*, and *KI67* gene expression^11^. Interestingly, all genes found to be overexpressed (ECM degradation signature) were correlated to mesenchymal markers and to basal-like/mesenchymal subtypes (triple-negative BC), which are associated with a higher risk of metastasis and poor prognosis. On the opposite, the *SCNN1A* gene was associated with normal breast or Luminal A BC and to the expression of epithelial markers. Moreover, we confirmed, in a cohort of 47 BC, an increased expression of *MMP9* and *ADAM19* in aggressive tumors as well as a decreased expression of *SCNN1A* (Supp Fig. 9A). Finally, a positive correlation could be established between *ADAM19* and *VIMENTIN* or *MMP9* and *VIMENTIN* in these samples (Supp Fig. 9B). These data confirmed that the regulation of genes involved in the degradation of ECM was not specific of a particular model, tissue, or pathology but seemed to be a reliable biomarker of EMT in different cancers in vivo.

### Characterization of ADAM19 as a new EMT marker in vivo

**Fig. 7:**
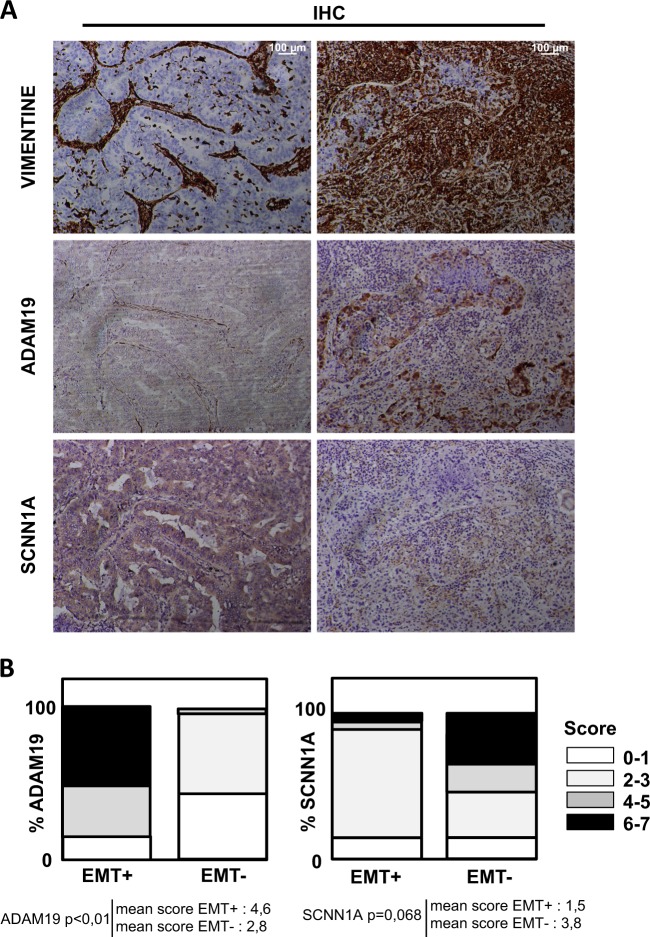
Immunohistological (IHC) staining of ADAM19 and SCNN1A in non-small cell lung cancer is associated with epithelial–mesenchymal transition (EMT) status. **a** Representative staining of IHC against VIMENTIN, ADAM19, and SCNN1A showing a positive correlation between ADAM19 and VIMENTIN expression levels and an inverse correlation between VIM and SCNN1A in 30 patients tumors classified as EMT-positive. **b** Top: quantifications of ADAM19 and SCNN1A staining in regard to EMT status (percentage of patients). Bottom: mean of IHC score in VIM+ and VIM tumors

To investigate ADAM19 expression in vivo in EMT, we quantified the expression of ADAM19 and SCNN1A in NSCLCs by immunohistochemistry (IHC) (Fig. 7). Biopsies were stained for VIMENTIN and 30 patients were sorted according to their levels of VIMENTIN: high (EMT+) or low (EMT−) VIMENTIN staining. These data confirmed that ADAM19 was overexpressed in EMT+ tissues and repressed in EMT− while SCNN1A expression was repressed in the EMT+ tissues and increased in EMT−.

## Discussion

Although epigenetic modifications have already been associated with EMT^12^, most of the studies were based on only one EMT model and one inducer. In order to characterize the global role of epigenetics during EMT, we performed an extended study on different EMT cell models (A549, ACHN, MCF10A). The transcriptome analysis revealed that most of the genes upregulated or downregulated in EMT were different in these models (downregulated in A549 alone: 1020/1497, in ACHN alone: 205/432, in MCF10A alone: 1433/1834 and upregulated in A549 alone: 652/1235, in ACHN alone: 178/507, in MCF10A alone: 1033/1647, Fig. 2b). The lower number of DE genes and the lower fold change of expression in the EMT-induced ACHN model compared to A549 and MCF10A suggested that TGFβ/TNFα signaling was less pronounced in ACHN.

These data suggested that EMT signaling could be, at least partially, cell-specific despite going through the same pathway of EMT induction. Indeed, ChIP-seq analysis in non-EMT models previously revealed that TGFβ-induced specific recruitment of SMAD3 on target genes was dependent of the cell type^13^. However, among these EMT-modulated genes, some seemed to be similarly regulated in our models (Supp Table 4 and Supp Table 5) and, in particular, the previously identified EMT markers *CDH1*, *EPCAM*, *VIMENTIN*, and *MMPs*. When comparing the list of the ten most upregulated genes in MCF10A and H358 treated with TGFβ/OSM (Oncostatin M) with our data, we observed that four genes (*GPR68*, *SERPINE1*, *ADAM19*, *SLAMF8*) were also found to be upregulated in our models and three additional genes (*FAP*, *MMP9*, *SCG5*) were upregulated in two out of three of our models following TGFβ/TNFα treatment^14^. Moreover, 9/10 of these genes were also previously reported as upregulated in the EMT-induced A549 model^15^. These data strongly suggested that few genes were similarly regulated in different EMT models. Moreover, *ADAM19* and *MMP9* were also highly expressed in the mesenchymal MDA-MB-231 cells and strongly increased in the EMT-induced MDA-MB-157 cells (Supp Fig. 7).

We also described several genes with high differences of expression between control and EMT-induced cells in the three models but that have never been related to EMT. For example, the expression of the poorly described *C15orf48* lncRNA, which has already been associated with cancer, was overexpressed (about 45-fold)^16^. *DHRS2*, overexpressed in the 3 cell models (about 38.9-fold) and encoding a dehydrogenase/reductase, has recently been associated with gastric carcinogenesis and 5-FU resistance^17^. On the opposite, the expression of *PPFIBP2* was downregulated in all our models (about 10-fold). Although very few data are currently available, *PPFIBP2* loci has been recently associated with increased probability of prostate cancer^18^. Altogether these data showed that all these genes might be considered as new markers of EMT in cancer.

We identified a specific and concomitant increase in positive, H3K4me2/3, and negative, H3K9me3 and H3K27me3, histone marks following TGFβ/TNFα treatment and we performed a ChIP-seq analysis targeting H3K4me2, with a profile similar to active genes in vertebrates^19^. We identified numerous genes whose regulation could be explained by epigenetic modifications on their promoters and we highlighted an epigenetic signature on genes involved in ECM remodeling, such as *MMP9* and *ADAM19*, which was increased in A549, ACHN, and MCF10A and also previously reported in another study^14^. This was associated with an increase in the H3K4me2 mark on their promoters during EMT. These results were consistent with a study showing that *MMP1/9/10* expression belonged to a gene cluster that overlaps an active enhancer^15^. We confirmed this analysis by showing a decrease of the H3K27me3 mark on the promoters of *MMP9* and *ADAM19*. Our data were in agreement with previously published results describing that the presence of these two latter marks were inversely correlated to one promoter^20^. To determine whether the epigenetic signature observed on genes involved in ECM degradation was specific of EMT, and not to TGFβ/TNFα treatment, we performed the same set of experiments on an independent model (MDA-MB-468 cells in which EMT was induced using EGF^21,22^). Indeed, we observed a progressive decrease in *CDH1* expression and an increase in *VIM* expression from day2. Similarly, a strong increase in expression of *SNAI1* was also observed at day 2 and then progressively decreased at day 3. The induction of SNAIL protein expression was only visible at day 1, suggesting that SNAIL expression is thinly regulated during EMT.

EMT induction in this model also led to a large overall epigenetic reprogramming, in particular in genes involved in ECM remodeling. Indeed, we detected an enrichment in H3K4me2 on the *ADAM19* promoter. But, interestingly, some of the genes involved in the ECM degradation presented a different pattern in the MDA-MB-468 (Supp Fig. 8). These results demonstrated that epigenetic programs and expression profiles linked to EMT differed according to the model and the cytokine treatment.

We then established a list of seven new biomarkers of EMT that presented a similar regulation in our different models of EMT together with an enrichment of H3K4me2 on their promoter (Supp Fig. 8C and D). In this list, we highlighted *MYO10* and *ADAM19*, two genes linked to tumor aggressiveness. *MYO10* encodes a member of the myosin superfamily, which has been associated with metastasis^23^. *ADAM19* encodes a metalloprotease that belongs to the ADAM family associated with cancer progression^24^. This protein has been associated with EMT in a glioblastoma model where its silencing by the *miR-145* inhibited EMT^25^. Interestingly, the epigenetic signature of these seven genes was identical whichever the cell model and the inducer used. We confirmed these in vitro data in a cohort of 100 NSCLCs. We first selected 30 EMT-positive tumors presenting a high VIMENTIN staining and confirmed that in vivo EMT was indeed correlated to an overexpression of the ADAM19 protein in NSCLCs. Interestingly, *ADAM19* was one of the few genes for which a strong upregulation was observed in our 5 different EMT models (5 different cell lines, 2 different EMT inducers) and also found in independent studies with similar or additional models^14,15^. Altogether these data strongly supported the role of ADAM19 in EMT. Finally, survival analysis of lung cancer patients using the *kmplot.com* software showed that a high expression of genes involved in ECM degradation, such as *ADAM19* (*p* = 3.3E−5), *ADAMTS6* (*p* = 0.00032), or *MMP9* (*p* = 0.04), was associated with a poor prognosis (Supp Fig. 10).

In conclusion, our data demonstrated, for the first time, an epigenetic signature of genes involved in ECM remodeling during EMT. Our results suggested that *ADAM19* expression and its epigenetic regulation could be considered as a robust new biomarker of EMT in vitro and in vivo. Moreover, our work suggested that novel anticancer therapies combining conventional drugs with epidrugs, targeting *ADAM19* expression, might be tested in future preclinical studies to reduce metastasis and aggressiveness.

## Materials and methods

### Transcriptome analysis and qRT-PCR

Total RNA was isolated from cells using the Tri Reagent (TR118, Molecular Research Center) according to the manufacturer’s instructions and RNA quality was controlled using the Experion Analysis kit (Biorad, France). Transcriptome analysis was performed on extracted RNA from A549, ACHN, and MCF10A cell lines treated or not (control) with TGFβ/TNFα. This analysis was performed on four RNA preparations for each cell line and condition. RNA integrity (RNA Integrity Number ≥ 8) was confirmed using an Agilent 2100 bioanalyzer (Agilent Technologies, France). Then transcriptome profiling was analyzed using the Agilent whole human genome 8 × 60 K microarray (kit G4851C, Agilent Technologies). Total RNA was labeled and hybridized according to the manufacturer’s recommendations using the LowInput QuickAmp Labeling Kit One-Color (Agilent Technologies, 5190–2305), Gene expression Hybridization Kit (Agilent Technologies, 5188–5242), RNA Spike-in one Color Kit (Agilent Technologies, 5188–5282), and washing buffer (Agilent Technologies, 5188–5327). Raw intensity data were log2-transformed and normalized (intra-array and inter-array scaling) using the GeneSpring GX software (Agilent Technologies), then gene expression were submitted to ArrayExpress repository under accession number “XXXXX.”

Probes were selected according to their level of expression (intensity above background in treated and/or control conditions) and to their absolute fold change (FCA ≥ 2) between treated and control conditions. For each cell line, unequal variance *t* tests (Welch *t* test) were performed to identify DE probes between treated and control samples. Adjusted *p* values were calculated by controlling the false discovery rate according to the Benjamini and Hochberg procedure. Probes were considered as significantly DE if the adjusted *p* value was <0.01. Venn diagrams were obtained using the http://biopuce.insa-toulouse.fr/ExperimentExplorer/venn/venn_graph.php software. Gene ontology was performed using the PANTHER software^26^.

Reverse transcription was performed in a mix containing M-MLV (12 U, Sigma-Aldrich, M-1302) reverse transcriptase, oligodT (0.25 µM, Eurogentec, Belgium), random hexamers (1.25 µM, C118A Promega, France), and total RNA (1.5 μg) according to the manufacturer’s instructions (Sigma-Aldrich). Quantitative PCR were performed as duplicate using the Step one Real-Time PCR system (Applied Biosystems, France) and the Power SYBR Green PCR Master Mix (Applied Biosystems) according to the manufacturer’s instructions. Primers used in our study were designed using the primer 3 software or according to Gemmill et al.^5^ and are listed in the Supp Table 8.

### Cell culture

The A549 (NSCLCC) cell line was obtained from Dr Christophe Borg (INSERM UMR1098, Besançon, France), the ACHN (metastatic renal cancer) cells were obtained from Dr Viviane Gnemmi (INSERM UMRS995, Lille, France)^27^, MCF10A (immortalized breast cells) cells were obtained from Philippe JUIN (INSERM U1232, Nantes, France), and the MDA-MB-468 (metastatic BC) cell line were obtained from Dr Christine Gilles (Laboratory of Tumor and Developmental Biology, Liège, Belgium). A549 and ACHN cells were grown in Dulbecco’s modified Eagle’s medium (DMEM) 1 g/l glucose (Dominique Dutscher, L0066, France) and MDA-MB-468 were cultured in RPMI 1640 medium. Both medium were supplemented with fetal bovine serum (5%) (Dominique Dutscher, S1810), penicillin–streptomycin (50 U/ml) (Dominique Dutscher, L0018), and amphotericin B (1.25 µg/ml) (PAA, P11–001, France). Cells were cultured at 37 °C in 5% CO_2_, and routinely used at 70–80% confluence. MCF10A were cultured in DMEM-F12 supplemented with 20 ng/ml EGF (Sigma-Aldrich, France), 100 ng/ml choleric toxin (Sigma-Aldrich), 10 µg/ml insulin (Sigma-Aldrich), 0.5 µg/ml hydrocortisone, and antibiotics as described above. Cells were cultured at 37 °C in 5% CO_2_ and routinely used at 70–80% confluence. When indicated, EMT was induced for 1–5 days using 4 ng/ml TGFβ (100–21, Peprotech, France) (ACHN, A549 and MCF10A) and 20 ng/ml TNFα (300–01 A, Peprotech) or 20–50 ng/ml EGF (MDA-MB-468).

### Epigenetics

#### Quantification of histone modifications

Histones were prepared from fresh cell pellets using the EpiQuik Total Histone Extraction Kit (OP-0006, Euromedex, France) and the protein were quantified using the Bradford Protein Assay (Biorad, 5000006). The efficiency of histone extraction was controlled using migration (see below) and detection of total histones by Coomassie staining and then WB using an anti-H3 antibody. Histone posttranslational modifications were quantified using the EpiQuik Histone H3 Modification Multiplex Assay Kit (P-3100, Epigentek) as per the manufacturer’s instructions. Each histogram corresponds to the mean of 2 independent experiments and each measure was obtained using a pool of 100 ng of total histones issued from 2 independent extractions (50 ng + 50 ng).

#### Chromatin immunoprecipitation

Chromatin was prepared using the truChIP*™* Chromatin Shearing Kit (520154, Covaris, France) according to the manufacturer’s instructions. Each sample was submitted to a 8 min sonicated using the M220 Covaris sonicator. ChIP were performed using the IP-Star Compact Automated System (Diagenode, Belgium) with the Auto iDeal ChIP-seq Kit for Histones (C01010171, Diagenode) and 1 µg of ChIP-grade antibody or IgG (IG07–2, P.A.R.I.S.). Libraries were prepared from 5 ng of ChIP DNA and Input DNA with the NEBNext® Ultra™ DNA Library Prep Kit for Illumina (E7370S, New England Biolabs, USA), according to the manufacturer’s instructions. From each library, 50 bp single reads were sequenced using an Illumina Hiseq 1500 system (Illumina). Reads were filtered according to their quality (*Q* Score ≥ 30) and adapter sequences were removed using Cutadapt^28^. Reads were aligned to the human genome (hg19) using the BWA (version 0.7.10). We obtained a mean of 21 million (min = 10 M, max = 48 M, sd = 6 M) uniquely mapped reads per sample. H3K4me2 differentially enriched regions (peaks) were identified using SICER-df (*g* = *w* = 200 bp). We considered two pairs of libraries: (1) TGFβ-/TNFα-treated condition versus its input control, (2) non-treated condition versus its input control. The basic strategy of SICER-df is to identify significant islands using SICER.sh in each of the two pairs, merge the two sets of significant islands, and then determine the significance of changes by comparing merged islands between the treated and the non-treated condition^29^. A region was considered significantly enriched between treated and non-treated conditions if the adjusted *p* value (Benjamini and Hochberg) was <0.01 and the FCA ≥ 2. We used the PAVIS software to annotate the H3K4me2 marks with an upstream/downstream interval of ±5 Kb from transcription start site^30^. Integration of transcriptome and ChIP-seq data was performed using BETA on the H3K4me2 differentially enriched regions^31^. A gene was considered as potentially regulated by the H3K4me3 mark if its rank product was <0.01. We then used the DeepTools suite to generate heat maps of read coverage^32^, and we used GSEA to search for enriched molecular signatures among genes^33^. A gene set was considered as significantly enriched if the Fisher exact test *q*-value was <0.001. Primers used for the ChIP validations were designed using the primer 3 software and listed in the Supp Table 8 and antibodies are listed in Supp Table 9.

### Western blotting

For the preparation of total protein extracts, cells were scraped, harvested and lysed in RIPA buffer (50 mM Tris-HCl, pH 8, 150 mM NaCl, 1% Triton X100, 0.5% DOCA, 0.1% sodium dodecyl sulfate (SDS)) supplemented with protease inhibitors (104 mM AEBSF, 1.5 mM pepstatin A, 1.4 mM E-64, 4 mM bestatin, 2 mM leupeptin, and 80 µM aprotinin) for 30 min on ice, then sonicated for 15 s, and centrifuged at 10,000 × *g* for 10 min at 4 °C. Proteins were quantified using the Bradford method and then proteins (25–40 μg) were separated on TGX acrylamide gels (1610172, Biorad) at 300 V for 30 min using and transferred onto Transblot turbo PVDF (1704157, Bio-Rad) membranes for 10–15 min with the Transblot turbo (1704150, Biorad) according to the manufacturer’s recommendations. Membranes were saturated in TBS-Tween 20 0.1% supplemented with 5% milk for 1 h and then incubated with primary antibodies (listed in Supp. Table 9) overnight at 4 °C. Membranes were washed 3 times with TBS-Tween 20 0.1% and incubated with secondary anti-rabbit or anti-mouse horseradish peroxidase conjugate antibody according to the manufacturer’s instructions (BI2407, BI2413C, P.A.R.I.S., France). The membrane was then washed 3 times with TBS-Tween 20 0.1% and incubated with Clarity Western Cl substrate (1705051, Biorad) and chemiluminescence was monitored using a Biorad ChemiDoc^TM^XRS+.

### Zymography

Cells were cultivated for 3 days in complete medium, with or without TGFβ/ TNFα, and then medium was replaced by serum-free medium containing or not the cytokines for 2 additional days. Five hundred µl of medium was concentrated using Pierce Concentrator, PES 30K MWCO (88502, Thermo Scientific) columns at 15,000 × *g* for 5 min. Samples were diluted in 1× non-reducing sample buffer (25 mM Tris-HCl, pH6.8, 0.8% SDS, 4% glycerol, 0.002% bromophenol blue) and separated in 7.5% SDS-polyacrylamide gel electrophoresis containing 4 mg/ml Gelatin. The gel was washed 2 times with washing buffer (50 mM Tris-HCl, pH 7.5, 2.5% Triton X-100, 5 mM CaCl_2_, 1 µM ZnCl_2_) for 30 min, rinsed for 10 min at 37 °C in incubation buffer (50 mM Tris-HCl pH7.5, 1% Triton X-100, 5 mM CaCl_2_, 1 µM ZnCl_2_) with agitation, and then incubated for 24 h at 37 °C in the incubation buffer with agitation. The gel was stained for 1 h in 40% methanol, 10% acetic acid, and 0.5% Coomassie blue, rinsed with water, and destained in 40% methanol and 10% acetic acid before quantification.

### Immunofluorescence

Cells were cultured for 24 h on coverslips and then fixed with cold methanol for 20 min at −20 °C and then blocked with Blocking solution (82007, Olinkbioscience) for 1 h at 37 °C. Incubations with primary antibodies were performed overnight at 4 °C, and then cells were rinsed 3 times with TBS-tween 0.1%. Incubations with secondary antibodies goat anti-rabbit and got anti-mouse AlexaFluor 488 or 555 (Life technologies) for were performed 1 h at 37 °C, and then cells were rinsed 3 times with TBS-tween, stained with DAPI (4’,6’-diamidino-2-phénylindole), and mounted using Fluoromount Aqueous Mounting Medium (F4680, Sigma Aldrich). Images were acquired with an Olympus FV1000 laser scanning confocal microscope (×63 objective).

### Flow cytometry

For membrane staining, cells were incubated with the corresponding antibody for 30 min at 4 °C, then washed with phosphate-buffered saline and centrifuged for 10 min at 300 × *g*. Ten thousands cells from each sample were evaluated for fluorescence detection using BD FACSCanto cytometer (Becton Dickinson) and analyzed with the FACS Diva software.

### Tissue samples, IHC, and immunostaining assessment

Paraffin-embedded tissue biopsies of NSCLCs were collected in collaboration with the Tissue Biobank of the University of Liege (Liege, Belgium). This protocol was approved by the Ethics Committee of The University Hospital of Liège. The initial diagnosis of each case was confirmed by experienced histopathologists. IHC analysis was performed with a standard protocol detailed previously and the primary antibodies listed in Supp. Table 9^34^. Following IHC, samples were classified into two groups: EMT-positive versus EMT-negative in regard to the intensity of VIMENTIN staining (score (0–3) and extent (0–3). As previously described^35^, the 2 scores were multiplied to obtain a global score ranged between 0 and 9. All immune-labeled tissues were evaluated by two experienced histopathologists.

### Statistics

Mean comparison were analyzed using Student's *t* test with the GraphPad Prism5 software. Correlation indices were measured using Spearman test calculated with the ImageJ software. Significant values are highlighted in bold in each figure. Retrospective transcriptome analysis was performed using the R software version 3.3.1, R Foundation for Statistical Computing, Vienna, Austria. Transcriptomes were built from Affymetrix series (GPL570 platform) after GC-RMA normalization. For BCs, 2830 samples were used from the following GEO series: GSE12276, GSE12790, GSE18931, GSE2109, GSE22035, GSE23720, GSE23994, GSE25407, GSE26910, GSE30010, GSE3744, GSE5764, GSE17700, GSE26639, GSE16446, GSE18864, GSE22513, GSE19615, GSE20685, GSE21653, GSE23177, GSE9195, GSE6532. Samples were classified with PAM50 method using 50 genes (genefu R package). For lung cancers, 802 samples were normalized from the following GEO series: GSE101929, GSE10245, GSE18842, GSE22047, GSE30219, GSE32496, GSE37745, GSE43580, GSE77803.

## Supplementary information

Supp Fig. S1-S10 revised

supplemental figure legends
