# Supplementary material for: EMT is associated with an epigenetic signature of ECM remodeling genes
Source: Cell Death Dis. 2019 Feb 27;10(3):205. doi: 10.1038/s41419-019-1397-4 (PMC6393505; doi:10.1038/s41419-019-1397-4)
Supplement: Supplementary file 1 — Supp Fig. S1-S10 revised [file 41419_2019_1397_MOESM1_ESM.pptx]

## Slide 1
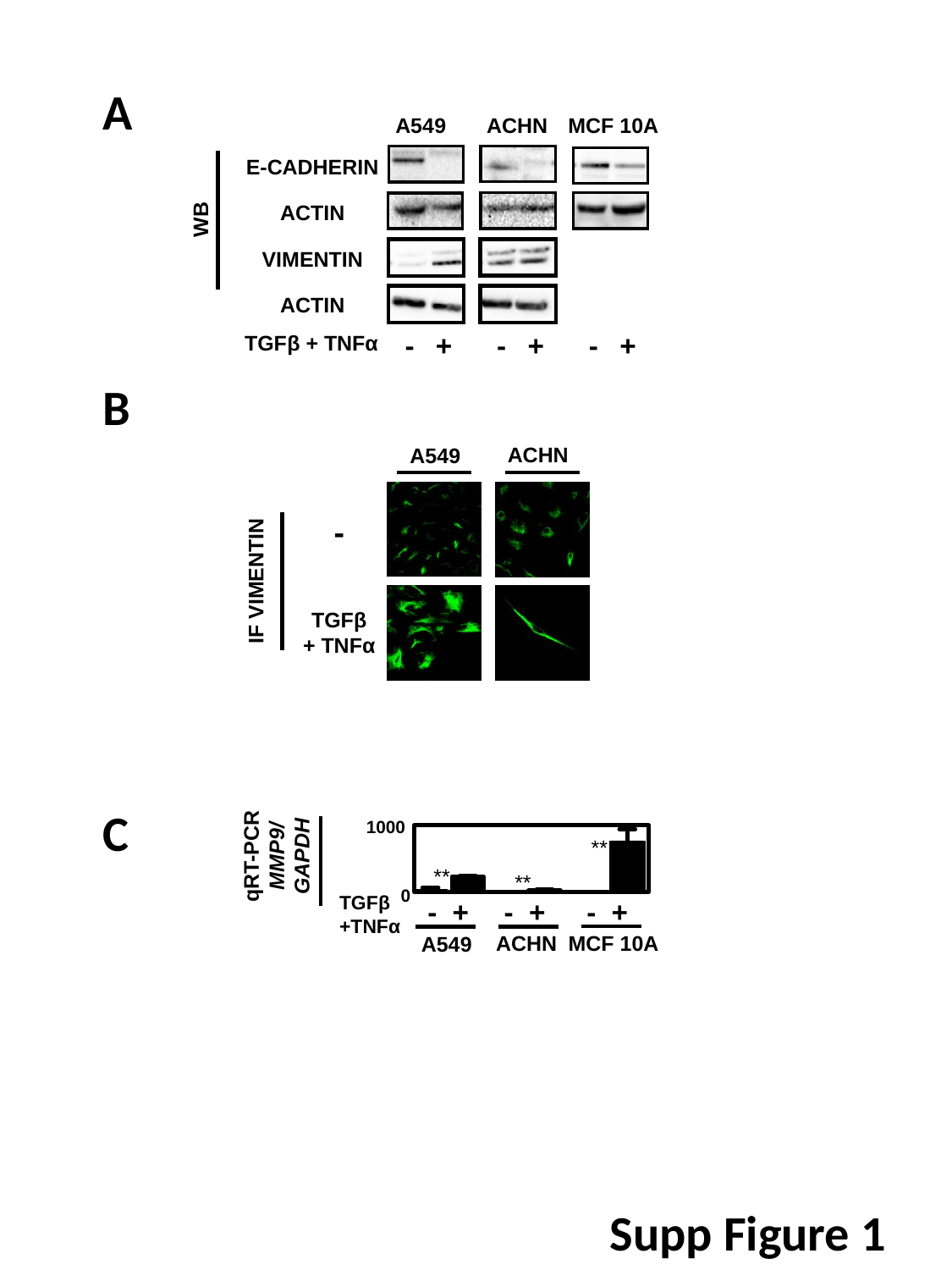

A
A549
ACHN
MCF 10A
E-CADHERIN
ACTIN
WB
VIMENTIN
ACTIN
TGFβ + TNFα
 - +
 - +
 - +
B
ACHN
A549
-
IF VIMENTIN
TGFβ
+ TNFα
C
1000
qRT-PCR MMP9/
GAPDH
**
**
**
0
TGFβ +TNFα
 - +
 - +
 - +
MCF 10A
ACHN
A549
Supp Figure 1

## Slide 2
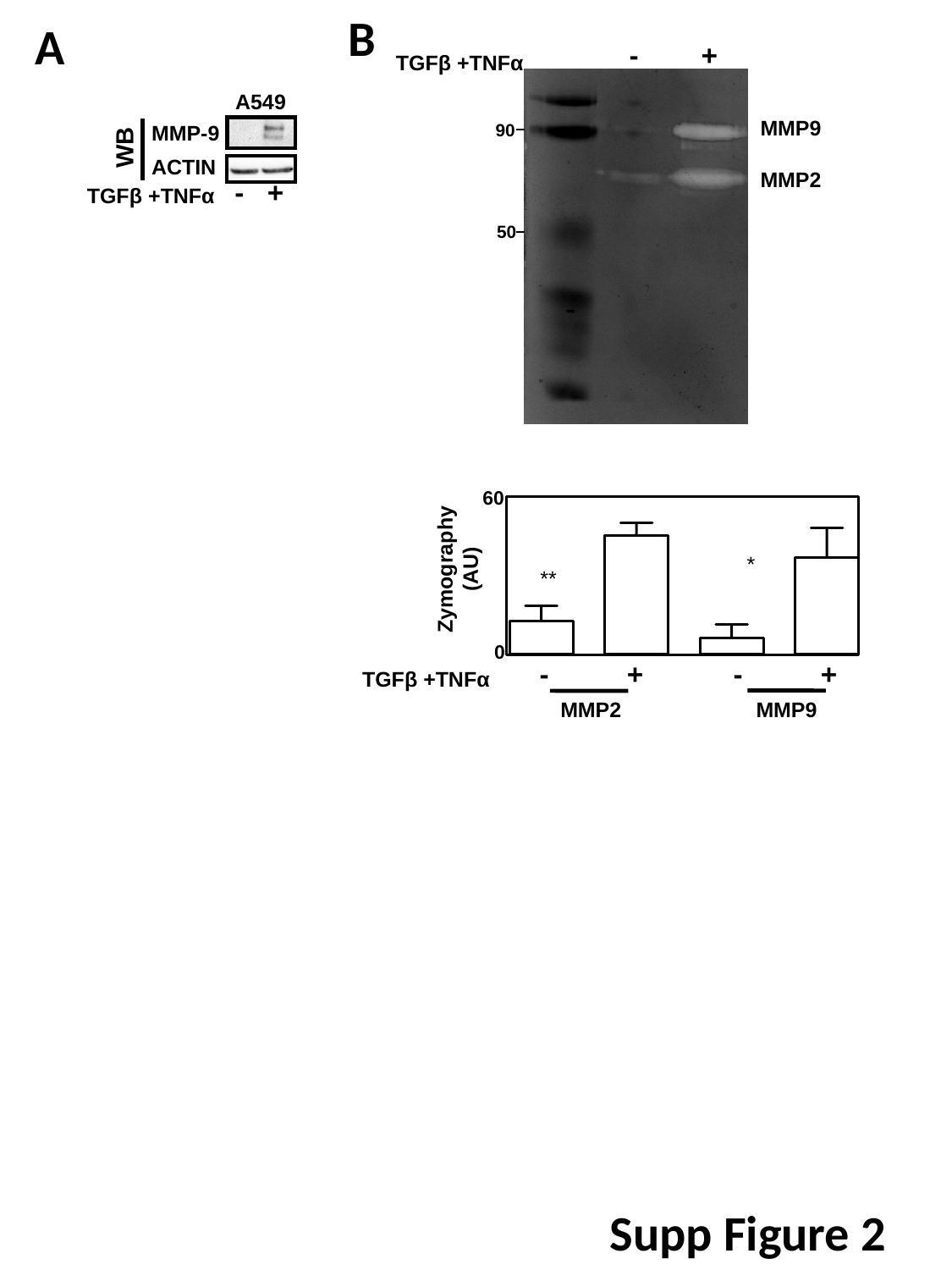

B
A
 - +
TGFβ
+TNFα
A549
MMP9
90
MMP-9
WB
ACTIN
MMP2
 - +
TGFβ
+TNFα
50
60
Zymography (AU)
*
**
0
 - +
 - +
TGFβ
+TNFα
MMP9
MMP2
Supp Figure 2

## Slide 3
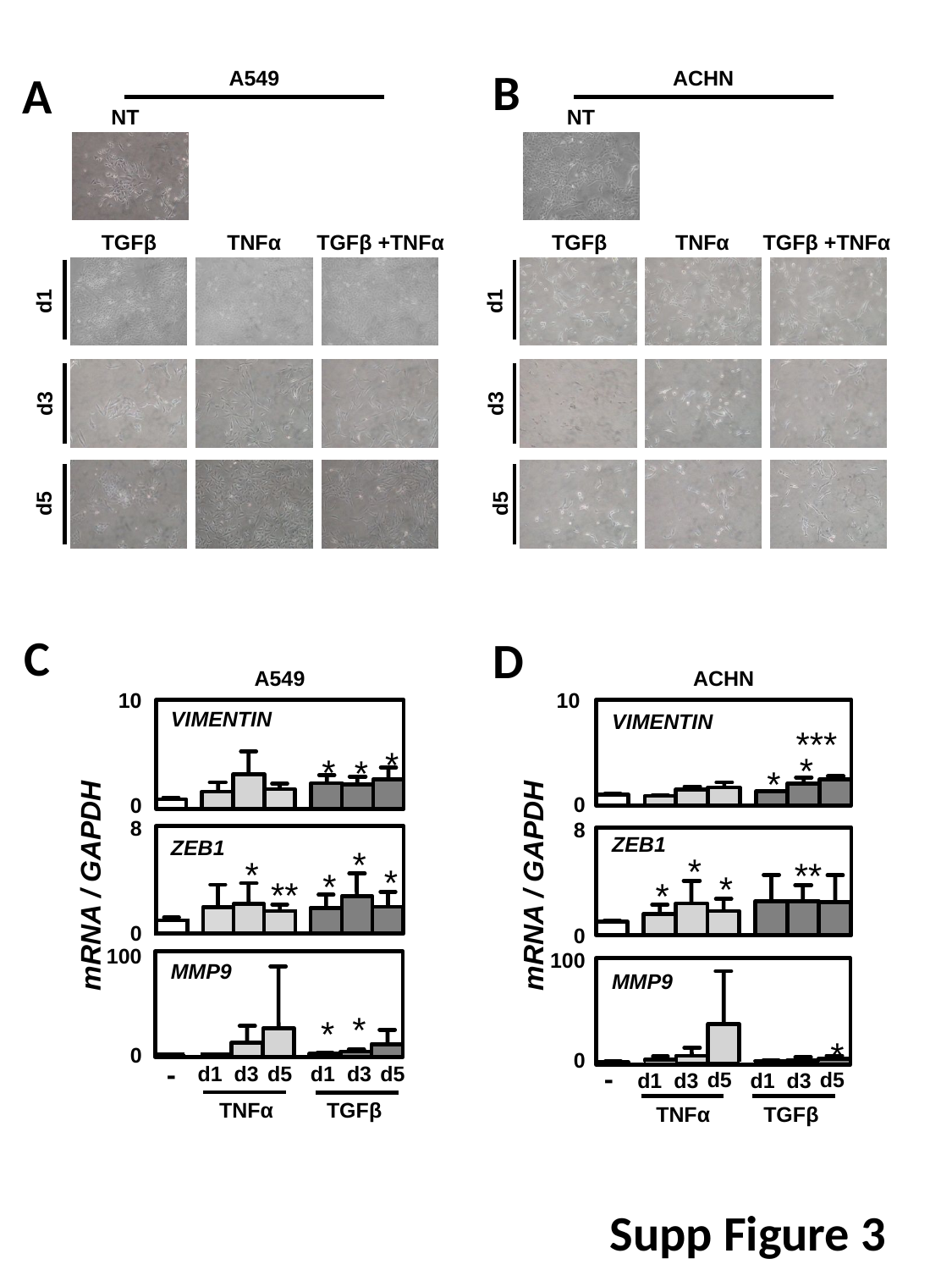

B
A549
ACHN
A
NT
NT
TGFβ
TNFα
TGFβ +TNFα
TGFβ
TNFα
TGFβ +TNFα
d1
d1
d3
d3
d5
d5
C
D
A549
ACHN
10
10
***
*
*
*
*
*
VIMENTIN
VIMENTIN
0
0
8
8
ZEB1
*
*
*
*
**
*
**
*
*
ZEB1
mRNA / GAPDH
mRNA / GAPDH
0
0
100
100
*
*
MMP9
*
MMP9
0
0
-
d5
d5
d3
d3
d1
d1
-
d5
d5
d3
d3
d1
d1
TNFα
TGFβ
TNFα
TGFβ
Supp Figure 3
Supp Figure 2

## Slide 4
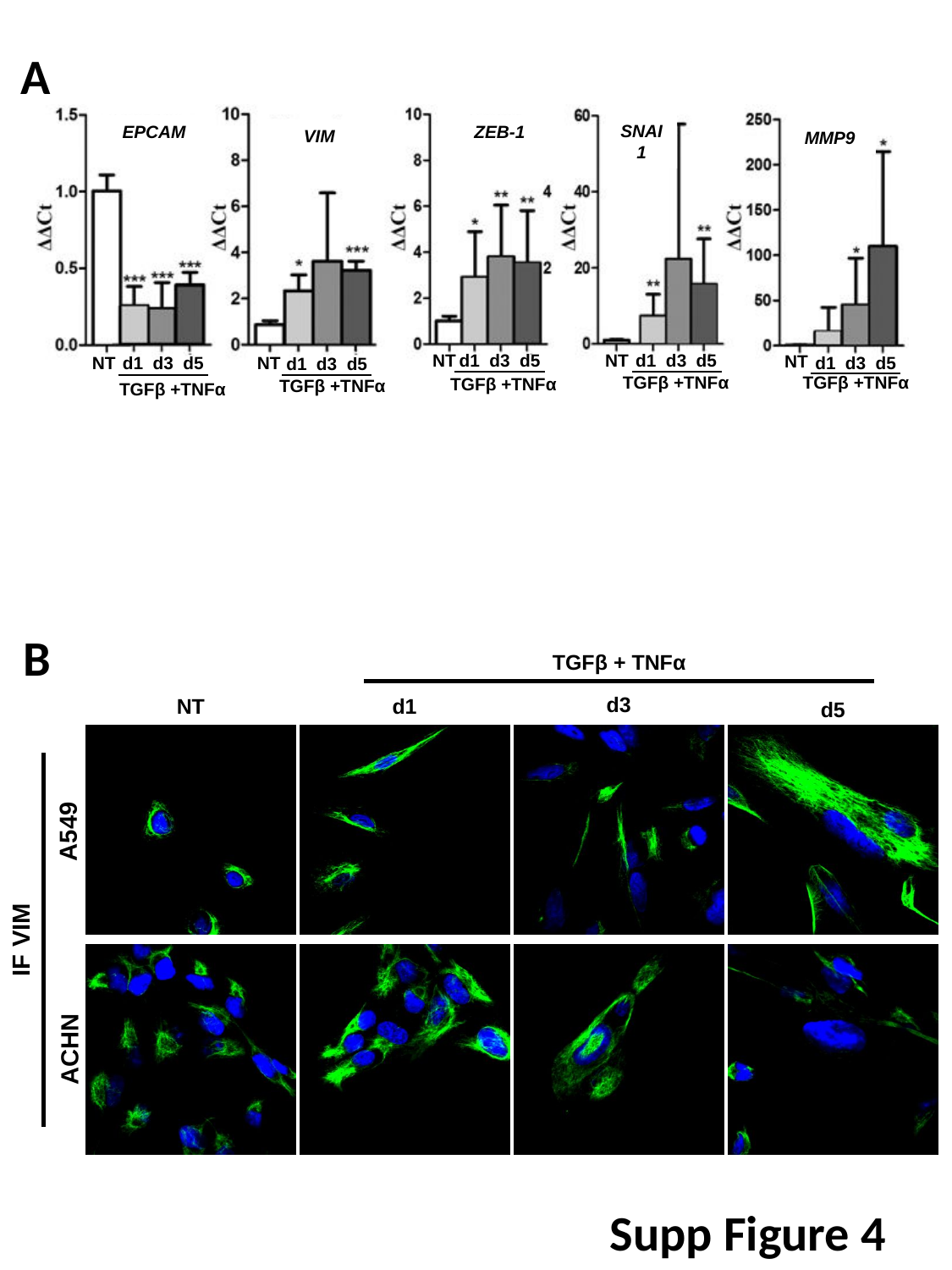

A
SNAI1
ZEB-1
EPCAM
VIM
MMP9
d1 d3 d5
NT
d1 d3 d5
NT
NT
d1 d3 d5
NT
NT
d1 d3 d5
d1 d3 d5
TGFβ +TNFα
TGFβ +TNFα
TGFβ +TNFα
TGFβ +TNFα
TGFβ +TNFα
B
TGFβ + TNFα
d3
NT
d1
d5
A549
IF VIM
ACHN
Supp Figure 4

## Slide 5
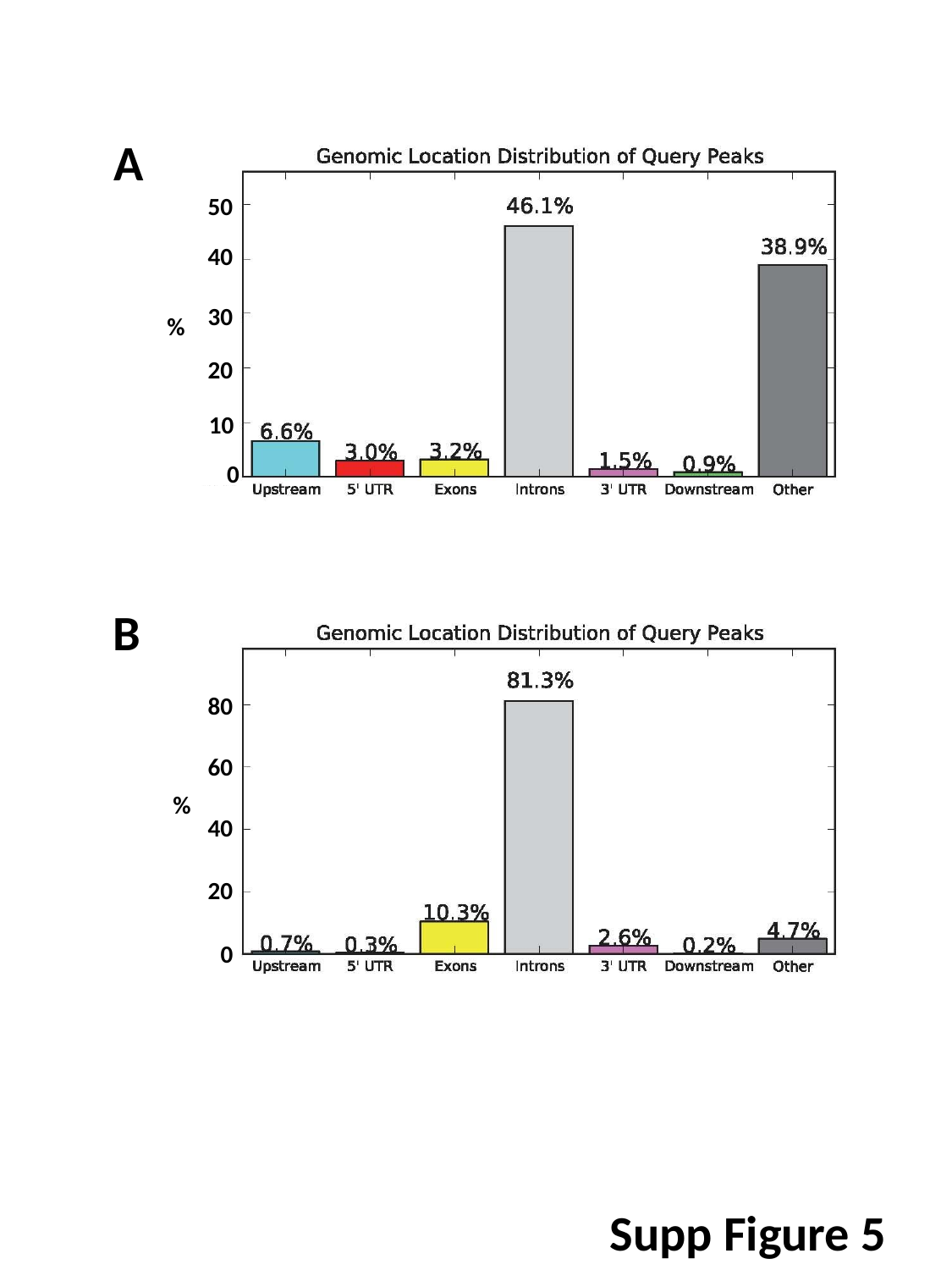

A
50
40
30
%
20
10
0
B
80
60
%
40
20
0
Supp Figure 5

## Slide 6
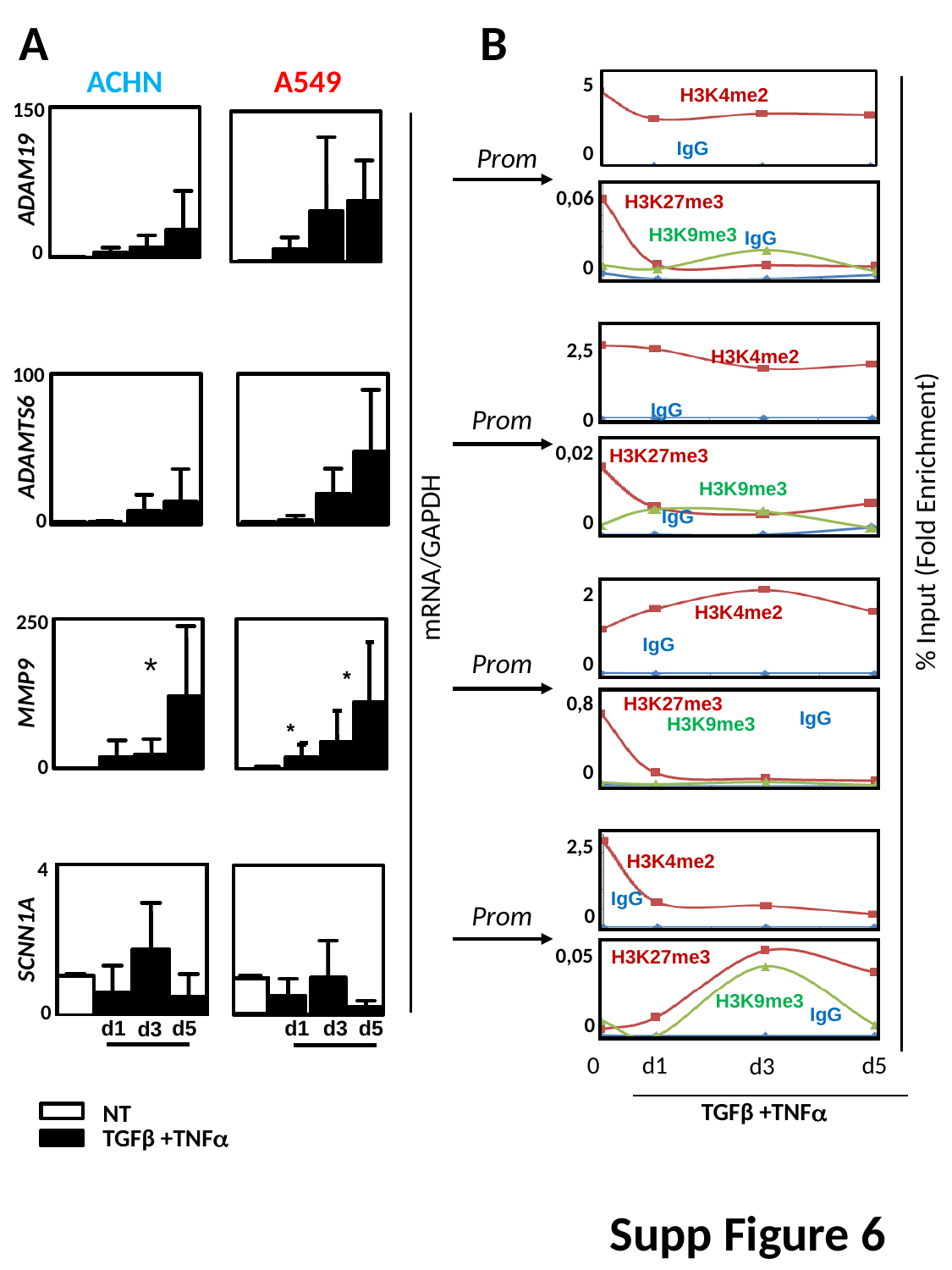

A
B
ACHN
A549
5
H3K4me2
IgG
0
H3K27me3
H3K9me3
IgG
0
150
0
Prom
ADAM19
0,06
2,5
H3K4me2
IgG
0
H3K27me3
0,02
H3K9me3
IgG
0
100
0
Prom
ADAMTS6
% Input (Fold Enrichment)
 mRNA/GAPDH
2
H3K4me2
IgG
0
H3K27me3
0,8
IgG
H3K9me3
0
250
0
Prom
*
*
MMP9
*
2,5
H3K4me2
IgG
0
H3K27me3
0,05
H3K9me3
IgG
0
4
0
Prom
SCNN1A
d5
d1
d3
d5
d1
d3
0
d1
d5
d3
TGFβ +TNFα
NT
TGFβ +TNFα
Supp Figure 6

## Slide 7
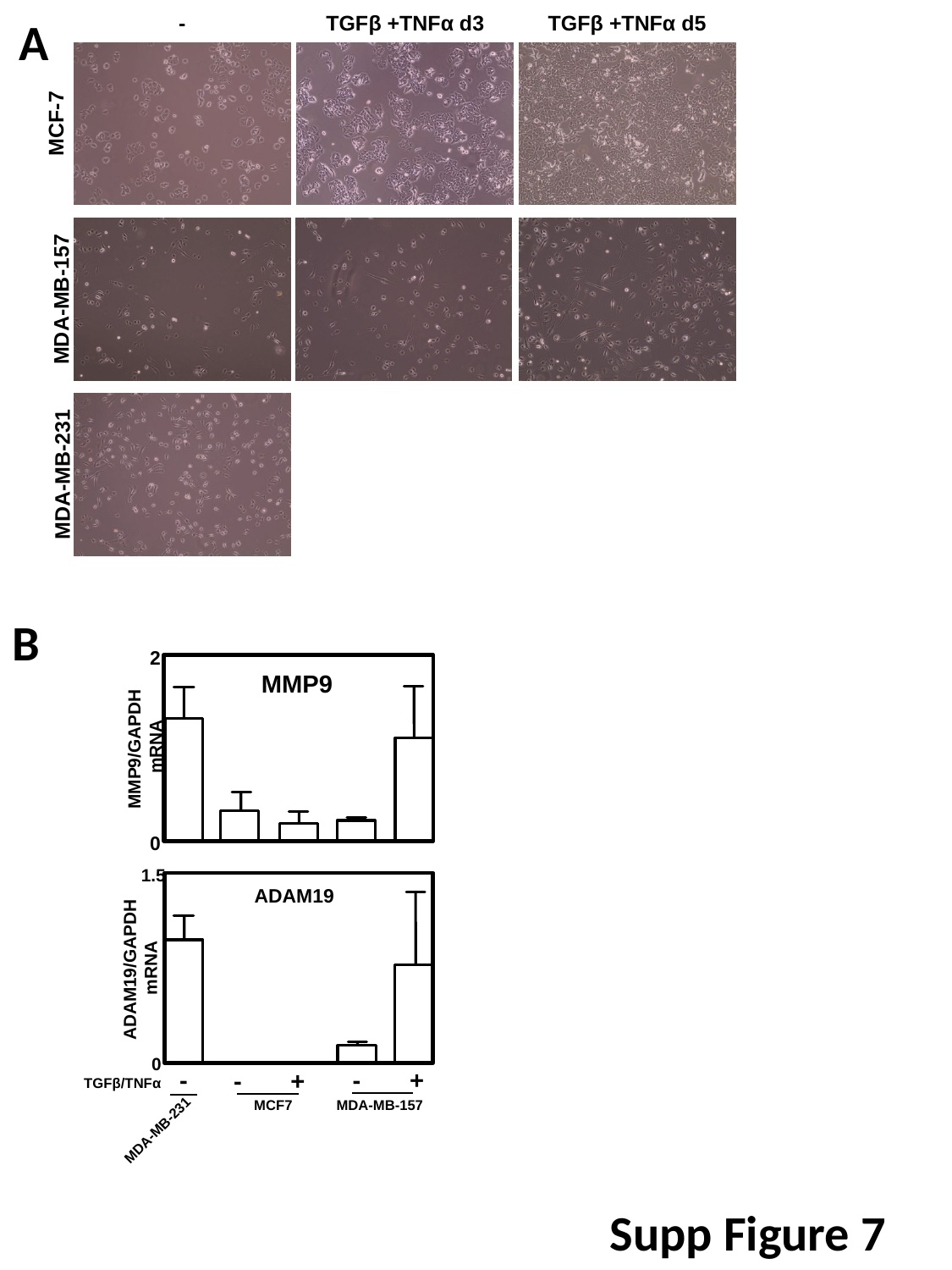

TGFβ +TNFα d5
-
TGFβ +TNFα d3
A
MCF-7
MDA-MB-157
MDA-MB-231
B
2
MMP9
0
MMP9/GAPDH
mRNA
1.5
ADAM19
ADAM19/GAPDH
mRNA
0
-
- +
- +
TGFβ/TNFα
MCF7
MDA-MB-157
MDA-MB-231
Supp Figure 7

## Slide 8
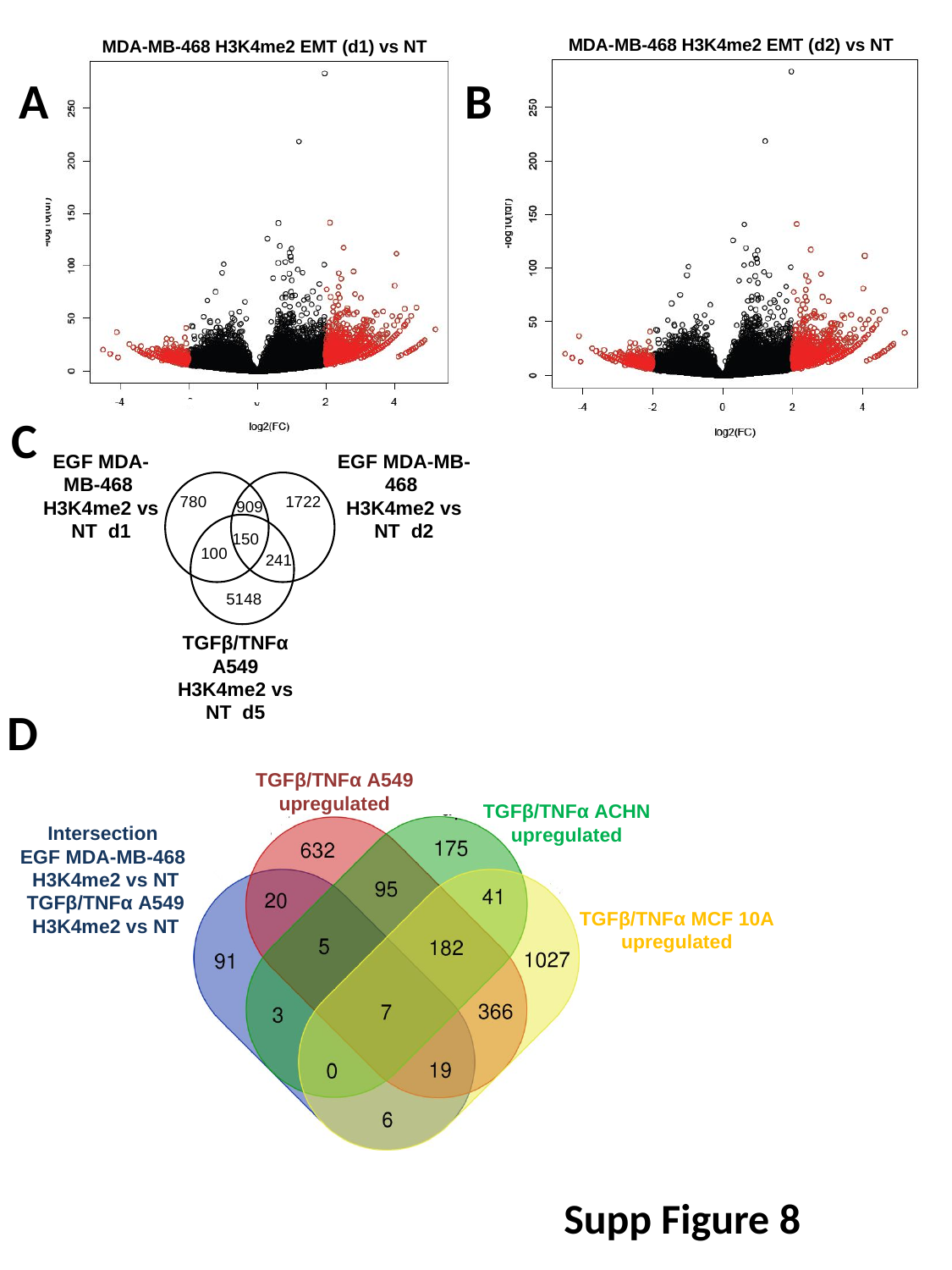

MDA-MB-468 H3K4me2 EMT (d2) vs NT
MDA-MB-468 H3K4me2 EMT (d1) vs NT
A
B
C
EGF MDA-MB-468
H3K4me2 vs NT d1
EGF MDA-MB-468
H3K4me2 vs NT d2
780
1722
909
150
100
241
5148
TGFβ/TNFα A549
H3K4me2 vs NT d5
D
TGFβ/TNFα A549
upregulated
TGFβ/TNFα ACHN
upregulated
Intersection
EGF MDA-MB-468
H3K4me2 vs NT
TGFβ/TNFα A549
H3K4me2 vs NT
TGFβ/TNFα MCF 10A
upregulated
Supp Figure 8

## Slide 9
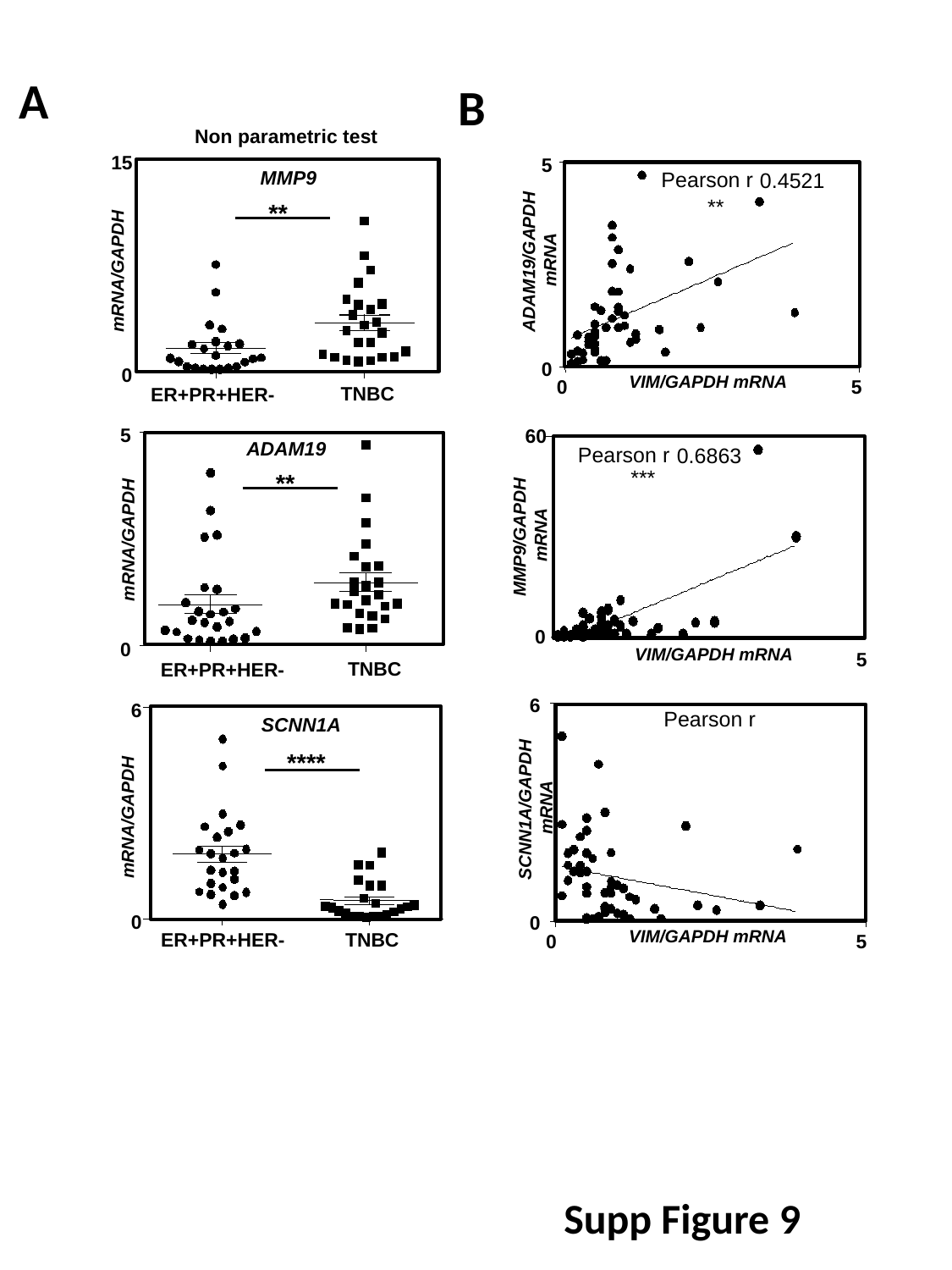

A
B
Non parametric test
15
**
0
TNBC
ER+PR+HER-
mRNA/GAPDH
5
ADAM19/GAPDH
 mRNA
0
0
5
VIM/GAPDH mRNA
Pearson r
0.4521
**
MMP9
5
0
ADAM19
**
mRNA/GAPDH
TNBC
ER+PR+HER-
60
0
Pearson r
0.6863
***
MMP9/GAPDH
 mRNA
VIM/GAPDH mRNA
5
6
0
0
5
6
0
SCNN1A
****
mRNA/GAPDH
TNBC
ER+PR+HER-
Pearson r
SCNN1A/GAPDH
 mRNA
VIM/GAPDH mRNA
Supp Figure 9

## Slide 10
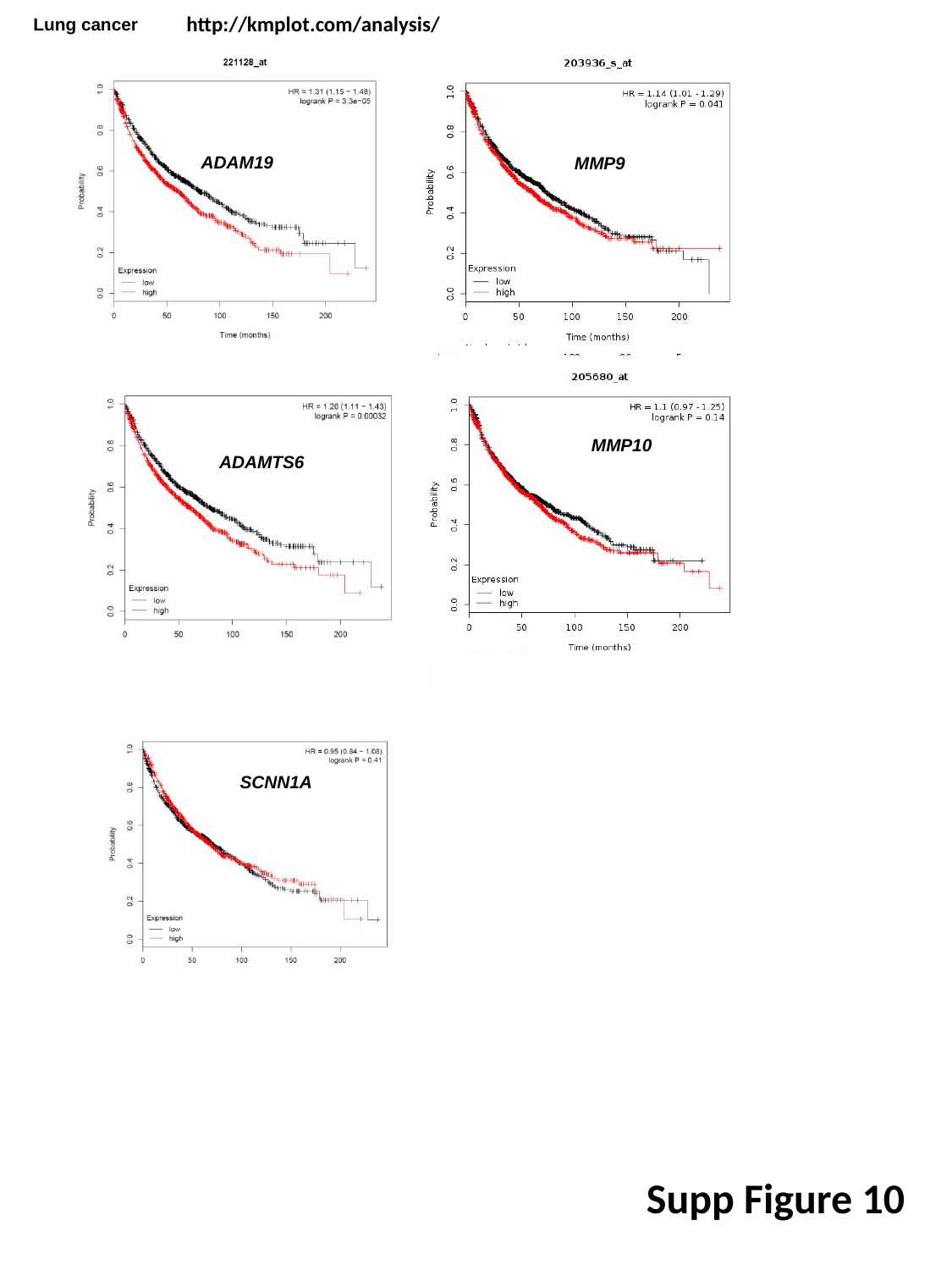

http://kmplot.com/analysis/
Lung cancer
ADAM19
MMP9
MMP10
ADAMTS6
SCNN1A
Supp Figure 10
