# Supplementary material for: EMT is associated with an epigenetic signature of ECM remodeling genes
Source: Cell Death Dis. 2019 Feb 27;10(3):205. doi: 10.1038/s41419-019-1397-4 (PMC6393505; doi:10.1038/s41419-019-1397-4)
Supplement: Supplementary file 2 — supplemental figure legends [file 41419_2019_1397_MOESM2_ESM.docx]

**Supp. Figure 1: Molecular markers of EMT are regulated by TGFβ/TNFα** **treatment.**

(**A**) Decreased expression of E-CADHERIN was confirmed using western blotting in A549 and ACHN cells following treatment with TGFβ/TNFα for 5 days. (**B**) An increase in VIMENTIN (green) staining was observed using IF in A549 and ACHN cells following treatment with TGFβ/TNFα for 5 days. Nuclei (blue) were stained with DAPI (**C**) Increased expression of *MMP9* by qRT-PCR in the 3 cell lines following treatment with TGFβ/TNFα for 5 days.

**Supp. Figure 2: Increase expression of MMP9 expression and activity in the EMT-induced A549 model.**

**(A)** MMP9 expression was quantified using WB in the A549 treated or not with the TNFα/TGFβ treatment. (B) Zymography confirmed increased activities of both MMP2 and MMP9 in media from EMT-induced A549 compared to control cells. Top: representative experiment. Bottom: quantification of 3 experiments.

**Supp. Figure 3: EMT induction was dependent of both TGFβ and TNFα** **treatment.**

A549 **(A)** and ACHN **(B)** cells were seeded in 6 multiwell dishes and treated for 5 days with TNFα, TGFβ or both cytokines. Pictures showing a progressive mesenchymal phenotype are representative of at least 3 independent treatments. Validation of EMT markers by qRT-PCR in A549 **(C)** and ACHN **(D)** cells.

**Supp. Figure 4: Modulation of expression of EMT markers is progressive during TGFβ/TNFα** **treatment.**

**(A)** A progressive increase of *VIM*, *ZEB-1*, *SNA1* and *MMP9* were measured by qRT-PCR after 1,3 or 5 days of TGFß/TNFα treatment. A stable decrease of the epithelial marker *EPCAM* was observed as soon at d1. **(B)** A progressive increase in VIMENTIN (green) staining was observed using IF in TGFβ/TNFα-treated A549 cells. Nuclei (blue) were stained with DAPI.

**Supp. Figure 5: Histograms presenting the genomic location distributions.**

(**A**) 1,952 significantly enriched H3K4me3 regions in TGFß/TNFα treated *vs.* non-treated conditions. (**B**) 614 upregulated genes potentially activated by the H3K4me3 mark.

**Supp. Figure 6: Kinetics of the regulation of gene expression in EMT.**

(**A**) Increased expression of *ADAM19, ADAMTS6, MMP9* and decrease expression in *SCNN1A* genes were quantified by qRT-PCR in the A549 and ACHN cells treated with or without TGFβ/TNFα for 5 days (mean of at least 3 independent experiments). (**B**) Quantification of H3K4me2, H3K9me3 and H3K27me3 modifications on the promoters of *MMP9*, and *SCNN1A* using ChIP in the A549 cells treated or not with TGFβ/TNFα for 5 days.

**Supp. Figure 7: Induction of ADAM19 in EMT in different BC models.**

(**A**) A mesenchymal phenotype was induced after with TGFβ/TNFα for 5 days in the MDA-MB-157 cells but not in the very epithelial MCF-7 cells. Mesenchymal MDA-MB-231 cells were used as a positive control. (**B**) Quantification of *MMP9* and *ADAM19* expression using qRT-PCR in the different BC models.

**Supp. Figure 8: ChIP-sequencing analysis on the H3K4me2 mark following EGF** **treatment.**

Volcano plot of the H3K4me2 merged islands in MDA-MB-468 cells treated respectively for 1 day (**A**) or 2 days (**B**) with EGF (vs NT). In red, the regions significantly enriched in EGF treated *vs.* non-treated conditions. FC: fold change treated *vs* non-treated. fdr: false discovery rate treated *vs* non-treated. (**C**) Venn diagram with H3K4me2 enriched genes in MDA-MB-468 cells treated with EGF for 1d, 2d or in A549 cells treated with TGFβ/TNFα. (**D**) Venn diagram with intersection of H3K4me2 enriched genes in MDA-MB-468 cells (treated with EGF for 1d, 2d) and A549 cells treated with TGFβ/TNFα (blue) *vs* genes up-regulated in A549 following TGFβ/TNFα treatment (red), *vs* up-regulated genes in ACHN following TGFβ/TNFα treatment (green), *vs* up-regulated genes in MCF 10A following TGFβ/TNFα treatment (yellow). 7 genes (including *ADAM19*) were up-regulated in the 3 TGFβ/TNFα treated models and enriched with the H3K4me2 mark in both TGFβ/TNFα treated A549 and in the EGF treated MDA-MB-468 cells.

**Supp. Figure 9: Signature of genes involved in ECM degradation in BC.**

(**A**) Expression of *MMP9*, *ADAM19* and *SCNN1A* were quantified using qRT-PCR in a cohort of 47 BC. (**B**) The correlation between the expression of these markers and *VIM* were analyzed using a Pearson-test.

**Supp. Figure 10: Kaplan-Meier curves of genes involved in ECM degradation.**

Kaplan-Meier using kmplot.com for gene expression of *ADAM19*, *ADAMTS6*, *MMP9*, *MMP10* and *SCNN1A* in lung cancer.

**Supp Table 1:** **Probes of differentially expressed in TGFß/TNFα-treated *vs* control conditions for the A549, ACHN, and MCF 10A cell lines).**

**Supp Table 2**: **Gene ontology based on up-regulated genes.** Pathways were identified using the PANTHER software.

**Supp Table 3: 1,952 H3K4me2 regions significantly enriched in TGFß/TNFα treated *vs.* non-treated conditions (1,740 up and 212 down-regulated) for the A549 cell line.**

**Supp Table 4: H3K4me2 regions associated with the 614 genes potentially activated by the H3K4me3 mark. GSEA of the 614 upregulated genes.**

**Supp Table 5: List of the 188 up-regulated genes following TGFß/TNFα in A549, ACHN and MCF 10A cells.**

**Supp Table 6: List of the 73 down-regulated genes following TGFß/TNFα in A549, ACHN and MCF 10A cells**

**Supp Table 7: List of the genes presenting a H3K4me2 enrichment in both TGFβ/TNFα-treated A549 and EGF-treated MDA-MB-468 cells**

**Supp Table 8: List of primers**

**Supp Table 9: List of antibodies**
